# Loss of NR2E3 represses AHR by LSD1 reprogramming, is associated with poor prognosis in liver cancer

**DOI:** 10.1038/s41598-017-11106-2

**Published:** 2017-09-06

**Authors:** Tilak Khanal, Kwangmin Choi, Yuet-Kin Leung, Jiang Wang, Dasom Kim, Vinothini Janakiram, Sung-Gook Cho, Alvaro Puga, Shuk-Mei Ho, Kyounghyun Kim

**Affiliations:** 1 Department of Environmental Health, University of Cincinnati, College of Medicine, 160 Panzeca way, Cincinnati, OH 45267 USA; 2 Division of Experimental Hematology and Cancer Biology, Cincinnati Children’s Hospital medical Center, Cincinnati, OH 45229 USA; 3 Department of Pathology and Laboratory Medicine, University of Cincinnati, College of Medicine, 231 Albert Sabin Way, Cincinnati, OH 45267 USA; 4 Center for Environmental Genetics (P30 ES006096), University of Cincinnati, College of Medicine, 160 Panzeca way, Cincinnati, OH 45267 USA; 5 Cincinnati Cancer Center, 231 Albert Sabin Way, Cincinnati, OH 45267 USA; 6 Department of Biotechnology, Korea National University of Transportation, Chung-ju, South Korea; 7 Cincinnati VA Medical Center, 3200 Vine Street, Cincinnati, OH 45220 USA

## Abstract

The aryl hydrocarbon receptor (AHR) plays crucial roles in inflammation, metabolic disorder, and cancer. However, the molecular mechanisms regulating AHR expression remain unknown. Here, we found that an orphan nuclear NR2E3 maintains AHR expression, and forms an active transcriptional complex with transcription factor Sp1 and coactivator GRIP1 in MCF-7 human breast and HepG2 liver cancer cell lines. NR2E3 loss promotes the recruitment of LSD1, a histone demethylase of histone 3 lysine 4 di-methylation (H3K4me2), to the AHR gene promoter region, resulting in repression of AHR expression. AHR expression and responsiveness along with H3K4me2 were significantly reduced in the livers of Nr2e3^rd7^ (Rd7) mice that express low NR2E3 relative to the livers of wild-type mice. SP2509, an LSD1 inhibitor, fully restored AHR expression and H3K4me2 levels in Rd7 mice. Lastly, we demonstrated that both AHR and NR2E3 are significantly associated with good clinical outcomes in liver cancer. Together, our results reveal a novel link between NR2E3, AHR, and liver cancer via LSD1-mediated H3K4me2 histone modification in liver cancer development.

## Introduction

Nuclear receptor subfamily 2 group E, Member 3 (NR2E3) is an orphan nuclear receptor that has been primarily characterized as a crucial player in retinal development^1–3^. Depending on the gene context, NR2E3 acts as either a transcriptional activator or repressor^4, 5^. However, the biological roles of NR2E3 in other tissues or diseases remain largely unknown. Previously, we reported that higher NR2E3 levels are strongly associated with good clinical outcomes in breast cancer by maintaining the expression of estrogen receptor α (ESR1), a major guideline for breast cancer prognosis and treatment^6^. We further revealed that environmentally induced oxidative stress decreased the level and homo-dimerization activity of NR2E3, resulting in a repressive epigenetic status of ESR1 gene promoter by LSD1 recruitment, consequently turning off ESR1 expression, indicating that NR2E3 is an oxidative stress-responsive epigenetic regulator^7^. Furthermore, ligand-activated potential of NR2E3 like other orphan nuclear receptors will provide opportunity to develop therapeutic targets in the future, although no ligand identified yet^8, 9^.

The AHR is a ligand-dependent basic helix-loop-helix transcription factor that can be activated by a broad spectrum of ligands, including environmental toxicants, potential endogenous ligands, and phytochemicals^10, 11^. Typically, after ligand binding, the AHR is translocated to the nucleus, forms a heterodimer with the AHR nuclear translocator (ARNT), and binds to the dioxin response element of target genes, including the CYP family of detoxification enzymes. The important roles of AHR in immunosuppression, metabolic diseases, and cancer development via crosstalk with multiple signaling pathways have been recognized by several recent studies^12–16^. Of note, loss of AHR facilitates tumor formation in several cancers, including the colon, prostate, and liver^17–19^, suggesting that AHR may exert tumor suppressive roles. Although AHR has been implicated in many human diseases and cancers, how AHR expression is maintained and regulated remains largely unknown.

Lysine-specific demethylase-1 (LSD1) is a flavin-dependent amine oxidase, which in general, functions as histone demethylase by removing the methyl group from mono- and dimethylated histone H3 at lysine 4 (H3K4), leading to suppression of the downstream target genes^19^. The genetic depletion of LSD1 in mice is known to cause embryonic lethality, and LSD1-depleted embryonic stem cells exhibit markedly reduced viability^20, 21^, indicating its crucial role in cell functions and survival. Many studies have shown that LSD1 overexpression increases cancer cell proliferation, invasion, and metastasis^22–28^. Furthermore, LSD1 overexpression is strongly correlated with poor clinical outcomes in many cancers, including liver cancer^29–31^. The inhibition of LSD1 activity with a small chemical inhibitor markedly decreased aggressive cancer cell phenotype and stem cell features, as a result of which LSD1 inhibition has attracted considerable attention as a novel therapeutic strategy for cancer treatment^32–35^. Although the LSD1-dependent H3K4me2 status change plays an important role in normal cell physiology and cancer progression, the epigenetic factor that modulates the distribution and function of LSD1 in maintaining normal epigenome remains to be identified.

Here, we reveal that NR2E3 is a novel upstream regulator of AHR. The presence of NR2E3 facilitates the formation of a transcriptionally active complex with specificity protein 1 (Sp1) and glucocorticoid receptor-interacting protein1 (GRIP1) in the proximal promoter region of the AHR gene whereas NR2E3 depletion markedly decreased active dimethyl-histone H3 lysine 4 (H3K4me2) marks by enhancing recruitment of the LSD1-associated repressor complex. This event decreased AHR expression and responsiveness. We further demonstrated that higher expression of NR2E3 or AHR in liver cancer patients is strongly correlated with good clinical outcomes. These findings indicated that interaction between NR2E3 and LSD1 plays a critical role in maintaining the normal epigenome and gene expression and that disruption of this interaction is associated with increased susceptibility and progression of liver cancer development.

## Results

### Association of NR2E3 with AHR-related gene networks

**Figure 1:**
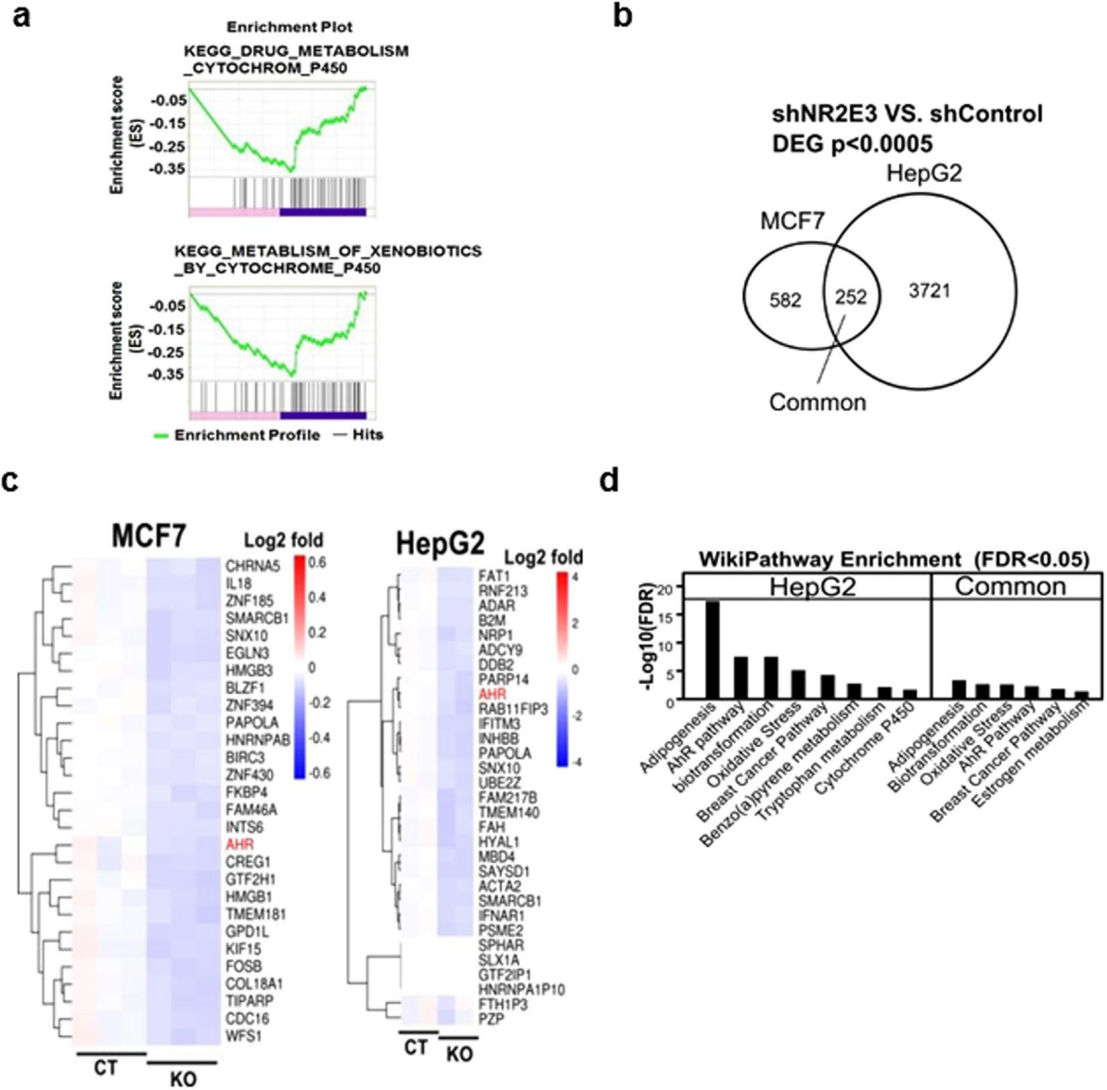
Identification of AHR as a novel target of NR2E3 by RNA sequencing (RNA-seq) and bioinformatics analyses. (**a**) Gene set enrichment analysis by employing RNA-seq data derived from small hairpin RNA control (shCT) and NR2E3-depleted (shNR2E3) HepG2 cell RNA lysate showed that NR2E3 gene networks are associated with drug metabolism involving cytochrome p450 and metabolism of xenobiotics by cytochrome p450 signaling pathways. (**b**) Venn diagram revealed 252 differentially expressed genes (DEGs) commonly regulated by NR2E3 depletion in both MCF-7 and HepG2 cells (p < 0.0005). (**c**) Heat map of the 253 common genes indicates that AHR expression (red) is markedly downregulated in both MCF-7 and HepG2 cells. (**d**) WikiPathway analysis using the common gene set showed the association of NR2E3-regulated genes with AHR-related signaling pathways (FDR < 0.05).

To identify crucial biological gene networks associated with NR2E3, we first obtained the NR2E3-dependent gene signature by performing RNA-seq analysis using small hairpin control (shCT) and NR2E3-depleted (shNR2E3) human liver HepG2 cell RNA lysates. A total of 3973 genes were differentially expressed by NR2E3 depletion (2,006 upregulated and 1,967 downregulated genes, with an adjusted P value [FDR q-value] < 0.05). GSEA analysis was carried out based on a ranked gene list and the results revealed that NR2E3 gene networks were significantly associated with AHR-related gene networks, including drug metabolism (cytochrome p450) and metabolism of xenobiotics (cytochrome p450; Fig. 1a and Supplementary Tables 1 and 2). These results are in line with the previous result suggesting that NR2E3 gene networks were closely related to AHR signaling pathways (GSE18431)^6^. Together with the previously obtained differential gene expression data derived from the control and NR2E3 knockout MCF-7 cell lysates, we identified the common gene set regulated by NR2E3 across two different cell types: ESR1-positive MCF-7 cells and ESR1-negative HepG2 cells. As seen in the Venn diagram, there are 252 commonly regulated genes (Fig. 1b and Supplementary Table 3) between the two groups. The heatmap indicated that AHR expression was commonly decreased in both NR2E3-depleted HepG2 and MCF-7 cell signatures (Fig. 1c). By using the common gene set, we also performed the WikiPathway analysis to identify the common biological pathways (Supplementary Tables 4 and 5), and the results consistently indicated that the NR2E3 gene networks are commonly highly associated with AHR signaling pathways (Fig. 1d).

### NR2E3 regulates AHR expression and responsiveness *in vitro*

**Figure 2:**
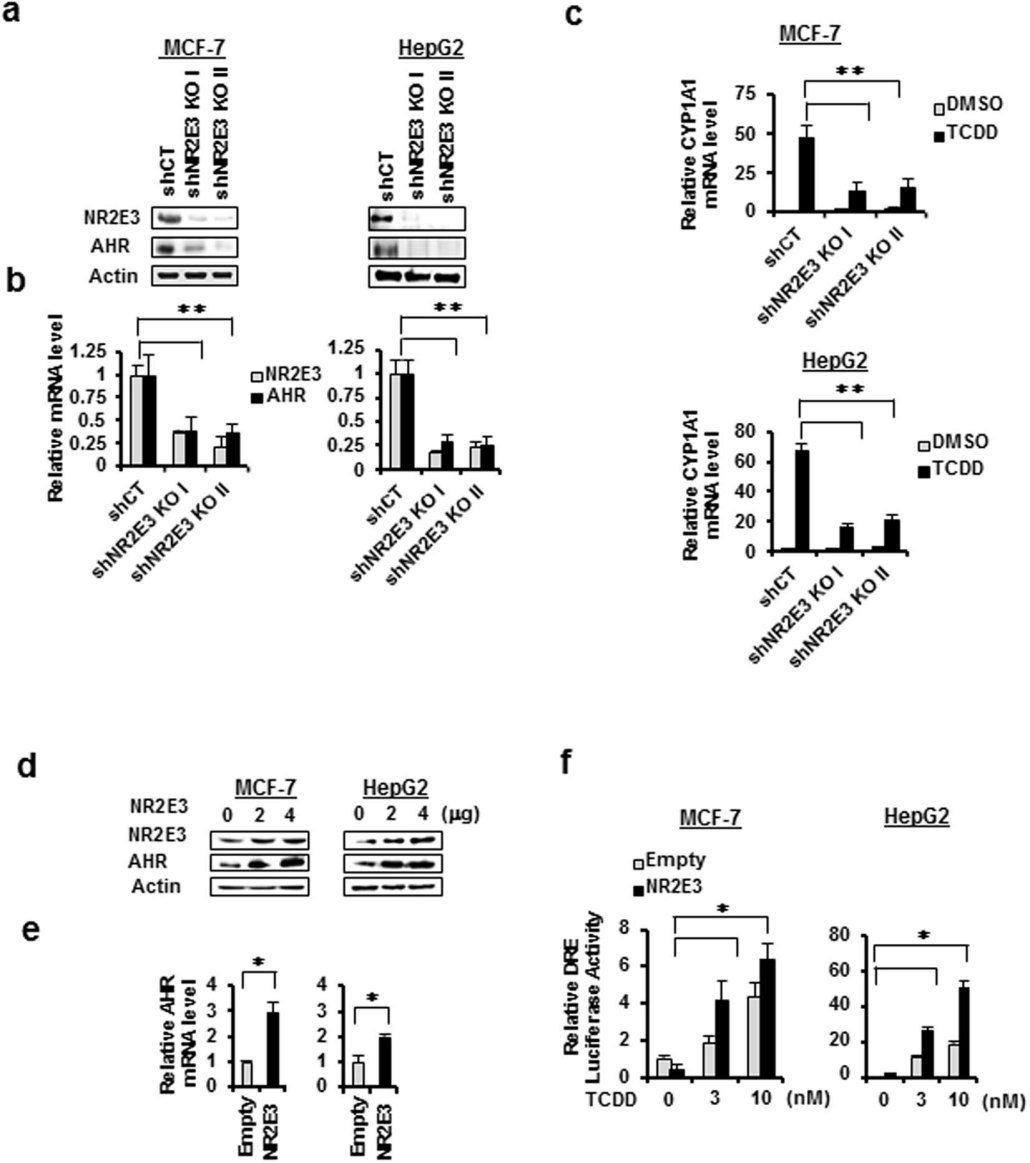
NR2E3 modulates AHR expression and responsiveness in cells. (**a**,**b**) MCF-7 and HepG2 cells were transfected with lentiviral-based small hairpin RNA (shRNA) targeting NR2E3 [shNR2E3 KO I & II] or a nonspecific control shRNA [shCT]. Effect of NR2E3 depletion on AHR and NR2E3 protein expressions and mRNA levels were detected by immunoblotting and qRT-PCR analysis. (**c**) Effect of NR2E3 depletion on the levels of CYP1A1 mRNAs induced by TCDD treatment (10 nM) in MCF-7 and HepG2 cells. (**d**,**e**) Effect of NR2E3 overexpression on AHR protein and mRNA levels in MCF-7 and HepG2 cells. (**f**) Effect of NR2E3 overexpression on the activity of reporter luciferase linked to consensus dioxin response element (DRE) in MCF-7 and HepG2 cells treated with and without TCDD treatment. Empty plasmid (Empty, pcDNA 4.0) was used as negative control. Results are means ± SE for at least two or three independent experiments with three replicates per experiment, and significantly (P < 0.05) increased (*) or reduced (**) responses are indicated.

Based on the strong biological association between NR2E3 and AHR, we studied the effects of NR2E3 depletion on the AHR expression level and responses. We silenced NR2E3 expression using shRNAs targeting NR2E3 in both MCF-7 and HepG2 cells. NR2E3 depletion markedly decreased both the AHR protein and mRNA expression levels in both MCF-7 and HepG2 cells (Fig. 2a and b). The results indicated that NR2E3 plays a role in maintaining AHR expression at the transcriptional level. Next, control (shCT) and NR2E3-depleted (shNR2E3) MCF-7 and HepG2 cells were treated with 2,3,7,8-tetrachloro-*p*-dibenzodioxin (TCDD), a typical ligand for AHR activation, and the induction level of CYP1A1, a major target gene induced by AHR activation, was then determined. The induction levels of CYP1A1 in NR2E3-depleted cells (shNR2E3 KO I & II) were greatly reduced compared to the levels in cells transfected with control scramble shRNA (shCT) (Fig. 2c). We then examined whether NR2E3 overexpression affected AHR expression levels. In this experiment, both MCF-7 and HepG2 cells were transiently transfected either with empty plasmid or NR2E3*-*expressing plasmid. NR2E3 overexpression significantly increased the AHR protein and mRNA levels (Fig. 2d and e). Consistently, the activity of reporter luciferase linked to DRE was greatly enhanced by NR2E3 overexpression when the cells were treated with TCDD (Fig. 2f). Taken together, our findings showed that NR2E3 maintains and positively regulates AHR expression at the transcriptional level, consequentially modulating its responsiveness.

### NR2E3 mediates transcriptionally active complex formation for AHR expression

**Figure 3:**
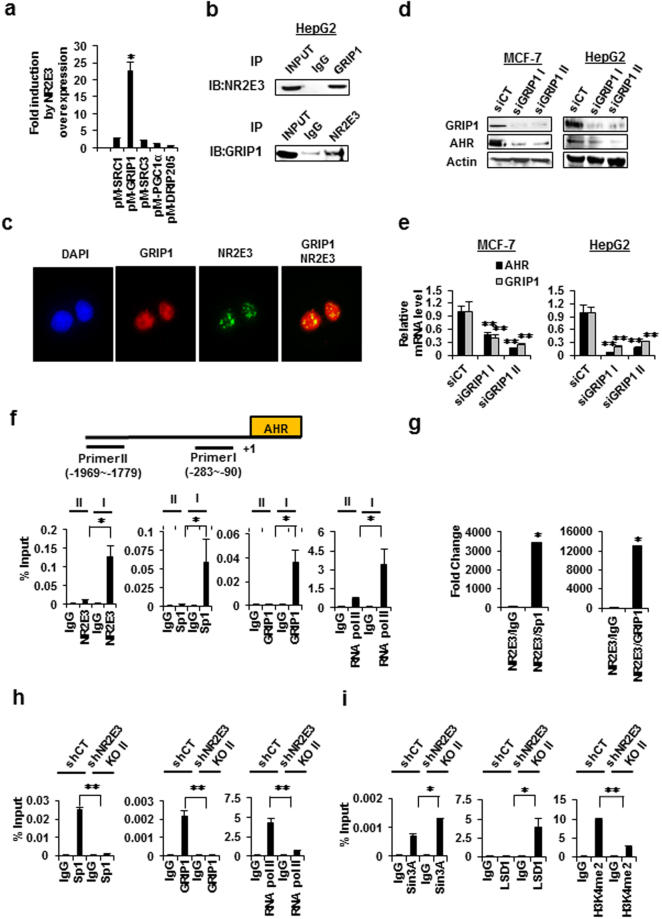
Formation of transcriptionally active complexes between NR2E3, Sp1, and GRIP1 on the proximal region of the AHR gene promoter. (**a**) Effects of NR2E3 overexpression on the GAL4 reporter luciferase activity in cells co-transfected with pM vector containing GAL4 DBD-linked coactivator, including SRC1, GRIP1 (SRC2), SRC3, PGC1α, and DRIP205 (TRAP220). (**b**) Co-IP assays confirmed the interactions between NR2E3 and GRIP1. HepG2 cells were lysed and Co-Immunoprecipitation (IP) was performed with anti-GRIP1 antibody or control non-immune IgG (IgG), followed by immunoblot analysis (IB) with NR2E3 antibody (Top). A reciprocal Co-IP assay with opposite set of antibodies was also performed (Bottom). (**c**) Co-localization of NR2E3 with GRIP1 in the nucleus of HepG2 cells. (**d**,**e**) Effect of GRIP1 depletion by using small interfering RNAs (siRNAs) targeting GRIP1 (siGRIP1 I and II) and scrambled control (siCT) on AHR and GRIP1 protein expression and mRNA levels in MCF-7 and HepG2 cells. (**f**) Binding of NR2E3, Sp1, and GRIP1 and RNA polymerase II on the AHR promoter regions, distal (−1969 to −1779) and proximal (−283 to −90) in HepG2 cells, as determined by ChIP-PCR. (**g**) Re-ChIP analysis of the complex formation between NR2E3 and Sp1 or NR2E3 and GRIP1 on the AHR proximal promoter region in HepG2 cells. (**h**,**i**) Effects of NR2E3 loss on the transcriptional and epigenetic status of the AHR proximal promoter. Binding of Sp1, GRIP1, RNA pol II, and H3K4me2 were reduced whereas binding of the corepressors Sin3A and LSD1 was increased. Results are means ± SE for at least two or three independent experiments with three replicates per experiment, and significantly (P < 0.05) increased (*) or reduced (**) responses are indicated.

We used the GAL4 reporter luciferase system to identify which coactivator plays a positive role in NR2E3-mediated AHR expression. Cells were transfected with plasmid containing GAL4 DNA-binding domain linked to various coactivators, including SRC1, GRIP1, SRC3, PGC1α, and DRIP205 (Trap220) and GAL4-reporter luciferase with or without NR2E3 overexpression. We found out that GAL4 luciferase activity was significantly enhanced only when cells were co-transfected with GAL4-GRIP1 and NR2E3 and not any other combinations (Fig. 3a), suggesting that NR2E3 specifically enhanced the coactivator function of GRIP1. To further determine whether NR2E3 forms protein complexes with GRIP1, co-immunoprecipitation assay was performed using HepG2 cell lysates. The reciprocal interactions between NR2E3 and GRIP1 were detected by co-immunoprecipitation assays and followed by immunoblotting with opposite set of antibodies (Fig. 3b top and bottom), demonstrating that endogenous NR2E3 formed the protein complex with GRIP1. We observed similar results when we used MCF-7 cell lysate (Supplementary Fig. 1a). To confirm whether anti-NR2E3 antibody pulled down NR2E3 protein, Co-immunoprecipitation assays were performed using both MCF-7 and HepG2 cell lysates (Supplementary Fig. 1b). In line with this result, immunocytochemical results showed that NR2E3 (green) and GRIP1 (red) were co-localized as multiple speckles within the nucleus (yellow) (Fig. 3c). In order to further define the role of GRIP1 in AHR expression, we examined whether GRIP1 depletion could affect AHR expression. MCF-7 and HepG2 cells were transfected with two types of siRNAs targeting different regions of the GRIP1 mRNA sequence (siGRIP1 I and II) or with scrambled control siRNA (siCT). GRIP1 depletion significantly decreased the AHR protein and mRNA levels (Fig. 3d and e), indicating that NR2E3/GRIP1 transcriptionally active protein complex formation is crucial for maintaining *AHR* expression. Several reports have previously demonstrated that specificity protein 1 (Sp1) transcription factor plays a positive role in maintaining AHR expression^36, 37^. Therefore, we examined whether NR2E3 also forms a protein complex with the Sp1 protein. The results of our study revealed that NR2E3 also interacts and co-localizes with Sp1 (Supplementary Fig. 2a and 2b). These results suggested that NR2E3, GRIP1, and Sp1 together form a protein complex.

We used ChIP-PCR to further verify whether NR2E3, GRIP1, and Sp1 form a complex on the AHR gene promoter region. We used a primer set covering the proximal region of the human AHR gene promoter (−283 to −90), which contains GC-rich Sp1-binding sites that was previously identified as a positive regulator for AHR expression^38, 39^. In contrast, the primer set that detects the distal region of the AHR promoter (−1969 to −1779) was used as a negative control (Fig. 3f, top). The results of the ChIP-PCR assays indicated that NR2E3 forms an active transcriptional complex with GRIP1 and Sp1 accompanied by RNA pol II recruitment (Fig. 3f, bottom) in the proximal region (Primer I) but not in the distal region (Primer II). Similar interactions were observed between NR2E3, Sp1, GRIP1, and RNA pol II in MCF-7 cells (Supplementary Fig. 2c). A Re-ChIP-PCR assay was performed to further confirm the NR2E3/GRIP1 or NR2E3/Sp1 protein complex formation. Formaldehyde-fixed chromatin was first immunoprecipitated with anti-NR2E3 antibodies followed by immunoprecipitation with anti-GRIP1 or anti-Sp1 antibodies. The corresponding immunoprecipitated lysate was used as a nonspecific control. The strong protein complex formed between NR2E3 and GRIP1 or NR2E3 and Sp1 in the proximal region of the AHR gene promoter region was then detected (Fig. 3g). Similarly, NR2E3/Sp1 and NR2E3/GRIP1 interactions were demonstrated by Re-ChIP-PCR assay using MCF-7 cell lysate (Supplementary Fig. 2d). These results demonstrated that NR2E3 is essential for the formation of active transcriptional complexes containing GRIP1 and Sp1 that are required in the maintenance of *AHR* expression.

Next, we investigated the effects of NR2E3 depletion on the epigenetic and transcriptional status of the AHR gene promoter, a ChIP-PCR assay was performed using NR2E3-depleted (shNR2E3 knockout I or II) and control HepG2 (shCT) cell lysates. NR2E3 depletion disrupted active protein complex formation of Sp1, GRIP1, and RNA pol II, and reduced their binding to this promoter region (−283 to −90; Fig. 3h). In contrast, binding of a corepressor SIN3A and a histone demethylase LSD1 to the proximal gene promoter of AHR was markedly increased, accompanied by reduced H3K4me2, which is an LSD1 substrate (Fig. 3i). Thus, NR2E3 loss not only disrupted the active transcriptional complex but also facilitated LSD1/Sin3A repressor complex recruitment. These results are in line with our previous report demonstrating that NR2E3 loss facilitates LSD1 recruitment and consequently decreased H3K4me2 on the proximal promoter region of the ESR1^7^.

### Effects of *Nr2e3* loss *in vivo* on *Ahr* expression and responsiveness

**Figure 4:**
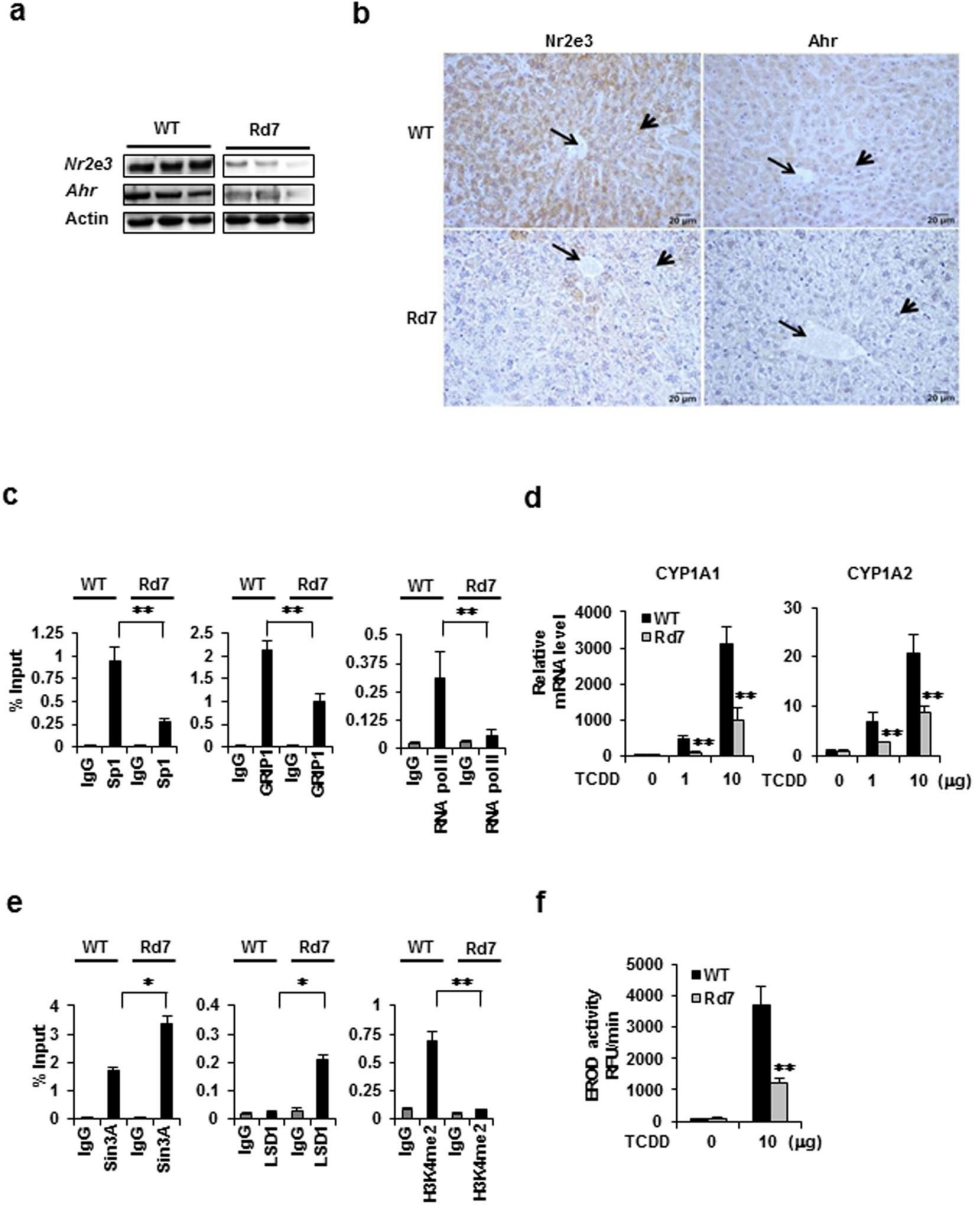
Effects of NR2E3 depletion on the transcriptional and epigenetic status of AHR gene promoter, AHR expression, and AHR-mediated responsiveness *in vivo*. (**a**) NR2E3 and AHR protein levels were determined using three individual wild-type (WT) and Nr2e3^rd7/rd7^ (Rd7) mice liver tissue lysates by immunoblotting. (**b**) Representative images of immunohistological staining of NR2E3 and AHR in WT and Nr2e3^rd7/rd7^ liver tissues (left) and with AHR (right). Long arrows: central vein; short arrows: pericental hepatocytes (**c**,**d**) Effects of NR2E3 loss on the transcriptional and epigenetic status of the AHR proximal promoter region in WT and Rd7 mice, as determined by *in vivo* ChIP-PCR analysis. (**e**) Significant decrease in the induction levels of CYP1A1 and CYP1A2 in Rd7 (N = 4) mice than in WT mice (N = 4; injected intraperitoneally with 0, 1, and 10 μg/kg of TCDD). (**f**) Measurement of ethoxyresorufin-*O*-deethylase (EROD) activity associated with CYP1A enzyme activities in TCDD-treated WT and Rd7 liver tissues. Results are means ± SE for at least two or three independent experiments with three replicates per experiment, and significantly (P < 0.05) increased (*) or reduced (**) responses are indicated.

To determine the role of NR2E3 in AHR status and AHR–mediated responsiveness *in vivo*, we employed *Nr2e3* ^Rd7/Rd7^ (retinal degeneration 7, Rd7) mutant mice (Jackson laboratory, Bar Harbor, MI) expressing non-detectable levels of *Nr2e3* in the retina^40, 41^. In Rd7 mice, the insertional mutation of LINE-1 transposon in the exon5 of *Nr2e3* gene inhibits proper *Nr2e3* mRNA processing and translation^41^. In fact, Rd7 mice have been primarily used to investigate the development of retinal diseases^1–4, 40–42^. As the liver is a major organ with high AHR activity for detoxification and metabolic processes, we examined whether the *Nr2e3* and *Ahr* expression levels were altered in the livers of Rd7 mice relative to the levels in the WT mice. We first identified that *Nr2e3* protein and mRNAs were expressed in the liver of WT mice (Fig. 4a and Supplementary Fig. 4c), unlike previous report^43^. Correspondingly, we observed significant decreases of both *Nr2e3* and *Ahr* protein and mRNA levels in the liver of Rd7 mice (Fig. 4a, Supplementary Figs 3a and 4a). In addition, with two more primer sets that amplify the region between exon 6 and exon 8 of mouse *Nr2e3* gene (mouse *Nr2e3* Exon 6-8 I & II), we further confirmed lower *Nr2e3* mRNA expressions in the liver of Rd7 mice (Supplementary Fig. 3a). In parallel, we investigated *Nr2e3* protein expression levels in the liver and retina of Rd7 mice by performing additional immunoblotting assay with another *Nr2e3* antibody (Abcam, Cat #172542). Results indicated that *Nr2e3* protein levels were low in the liver of Rd7 mice (Supplementary Fig. 4a and 4c) but not detectable in the retina (Supplementary Fig. 4b and d). Consistent with these findings, immunostaining results revealed considerably lower *Nr2e3* and *Ahr* levels in the livers of Rd7 mice (Fig. 4b). Intriguingly, we detected a much lower expression of *Esr1*, which is a previously identified target of *Nr2e3* ^6, 7^, in Rd7 mouse livers, but no change in the LSD1 level (Supplementary Fig. 3b and c), suggesting that *Nr2e3* plays a role in maintaining the *Ahr* and *Esr1* expression levels. In addition, we examined whether other *Ahr* downstream target genes were downregulated in Rd7 mice, and found out that the expression level of *Ppara*, an *Ahr* target gene that is downregulated in the livers of *Ahr* knockout mice, was unchanged^44^ (Supplementary Fig. 3d). Also, results from alanine aminotransferase (ALT) activity that typically detects liver injury showed no apparent liver damage in Rd7 mice (Supplementary Fig. 3e). *Nr2e3* likely regulates a different subset of gene networks that does not overlap with *Ahr* gene networks; moreover, *Nr2e3* loss did not cause any spontaneous liver injury *in vivo* (Supplementary Fig. 3e).

To examine whether *Nr2e3* loss could induce similar epigenetic and transcriptional status changes in the mouse *Ahr* gene promoter region, we performed *in vivo* ChIP assay using liver lysates of WT and Rd7 mice with the primer set covering the proximal promoter region (−138 to + 141). As expected, NR2E3 depletion resulted in significant disruption in the protein complex interactions between Sp1, GRIP1, and RNA pol II in this region (Fig. 4d) while Sin3A and LSD1 recruitment were enhanced, leading to decreased H3K4me2 levels (Fig. 4e). The patterns of epigenetic and transcriptional status changes induced by NR2E3 depletion were similar both *in vivo* and *in vitro*.

In order to determine whether ligand-activated *Ahr* responsiveness and downstream target gene expressions were altered by *Nr2e3* depletion, both WT and Rd7 mice were treated with TCDD, and the induction levels of *Cyp1a1* and *Cyp1a2*, which are the main target genes of ligand-activated *Ahr*, were then determined. The *Cyp1a1* and *Cyp1a2* mRNA levels were significantly lower in Rd7 mice than in WT mice (Fig. 4f). Consistently, significantly lower induction of *Cyp1b1*, another *Ahr* target gene, was observed (Supplementary Fig. 5). Correspondingly, significantly lower 7-ethoxy-resorufin-O-deethylase (EROD) activity, which is an indicator of *Cyp1a1* enzymatic activity, was detected in the livers of Rd7 mice relative to that in WT mice (Fig. 4f). These data demonstrated that *Nr2e3* depletion *in vivo* reduced *Ahr* expression by inducing repressive epigenetic and transcriptional status of the *Ahr* gene promoter in an LSD1-dependent manner via H3K4me2 modification. Consequently, we observed markedly reduced induction levels of *Cyp1a1* and *Cyp1a2* mRNAs and reduced enzymatic activities of these two enzymes.

### Restoration of AHR expression and H3K4me2 status by LSD1 inhibition *in vivo*

**Figure 5:**
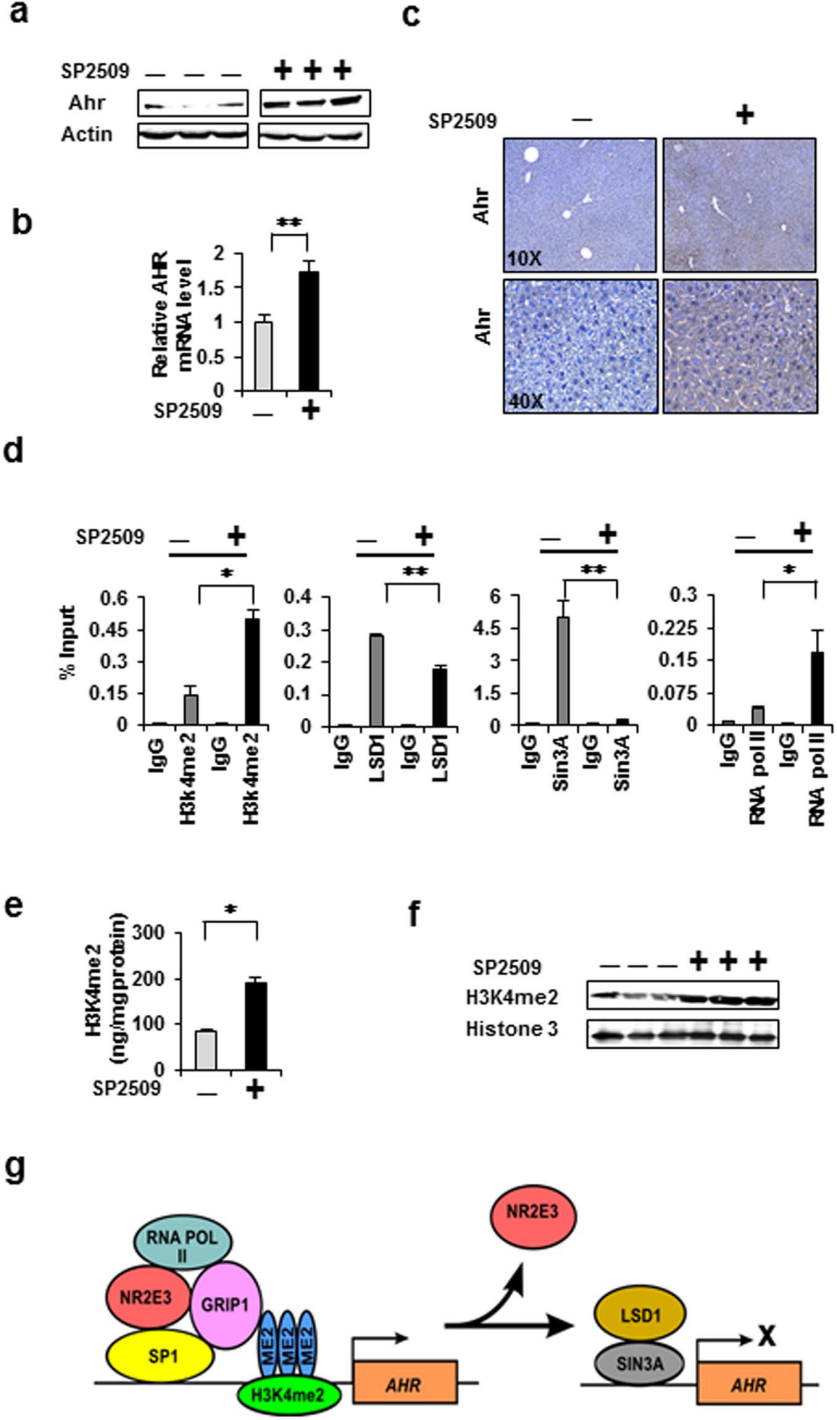
Restoration of AHR expression by administration of a chemical inhibitor of LSD1 to RD7 mice. (**a**,**b**) Rd7 mice were intraperitoneally injected with 15 mg/kg of SP2509 (twice with a 24-h interval). After 48 h, the livers were harvested, homogenized, and the AHR protein levels were analyzed by immunoblotting. AHR mRNA levels were determined by qRT-PCR (**c**) Representative images of immunostaining of Rd7 mice liver tissues treated with and without SP2509. (**d**) Effects of NR2E3 loss on the transcriptional and epigenetic status of the AHR proximal promoter region in Rd7 mice liver tissues treated with and without SP2509, determined by *in vivo* ChIP-PCR. (**e**) A proposed model for the role of NR2E3 in the transcriptional and epigenetic status of the AHR promoter. Results are means ± SE for at least two or three independent experiments with three replicates per experiment, and significantly (P < 0.05) increased (*) or reduced (**) responses are indicated.

Our results indicated that NR2E3 depletion in cells and mice facilitated the recruitment of LSD1, which greatly reduced the H3K4me2 histone marks in the AHR gene promoter (Figs 3 and 4). Therefore, to determine whether the histone demethylase activity of LSD1 plays a critical role in repressing AHR expression, we examined whether LSD1 inhibition could restore *Ahr* expression *in vivo*. We treated Rd7 mice with a chemical inhibitor of LSD1, SP2509^34, 45^. This treatment (i.p., 15 mg/kg) restored *Ahr* protein and mRNA expression in the livers of Rd7 mice (Fig. 5a and b); this result was verified by immunostaining analysis (Fig. 5c). Moreover, increased *Esr1* but not LSD1 expression was observed in Rd7 mice treated with SP2509 (Supplementary Fig. 6a and 6b). Both the *Ahr* and *Esr1* genes were repressed by the LSD1 redistribution induced by *Nr2e3* depletion (Figs 3I and 4e, Supplementary Fig. 3a–c). However, SP2509 treatment did not result in any changes in the AHR mRNA and protein levels in WT mice (Supplementary Fig. 7), suggesting that these genes are not normally repressed by LSD1.

A ChIP assay was performed to further validate the effects of SP2509 treatment on the epigenetic status change of the *Ahr* gene promoter. The active histone mark H3K4me2 and RNA pol II recruitment were increased while Sin3A binding was reduced with minimal change in the LSD1 binding status (Fig. 5d). Correspondingly, the overall H3K4me2 levels were significantly increased following SP2509 treatment; this increase was confirmed by measuring the H3K4me2 levels and by immunoblotting analysis (Fig. 5f). These results clearly demonstrated that the repression of *Ahr* expression is dependent on the histone demethylase of LSD1. We proposed a model to illustrate how NR2E3 loss alters the active epigenetic and transcriptional status of the AHR gene promoter to repressive state (Fig. 5g).

### Clinical association of AHR and NR2E3 with liver cancer development

**Figure 6:**
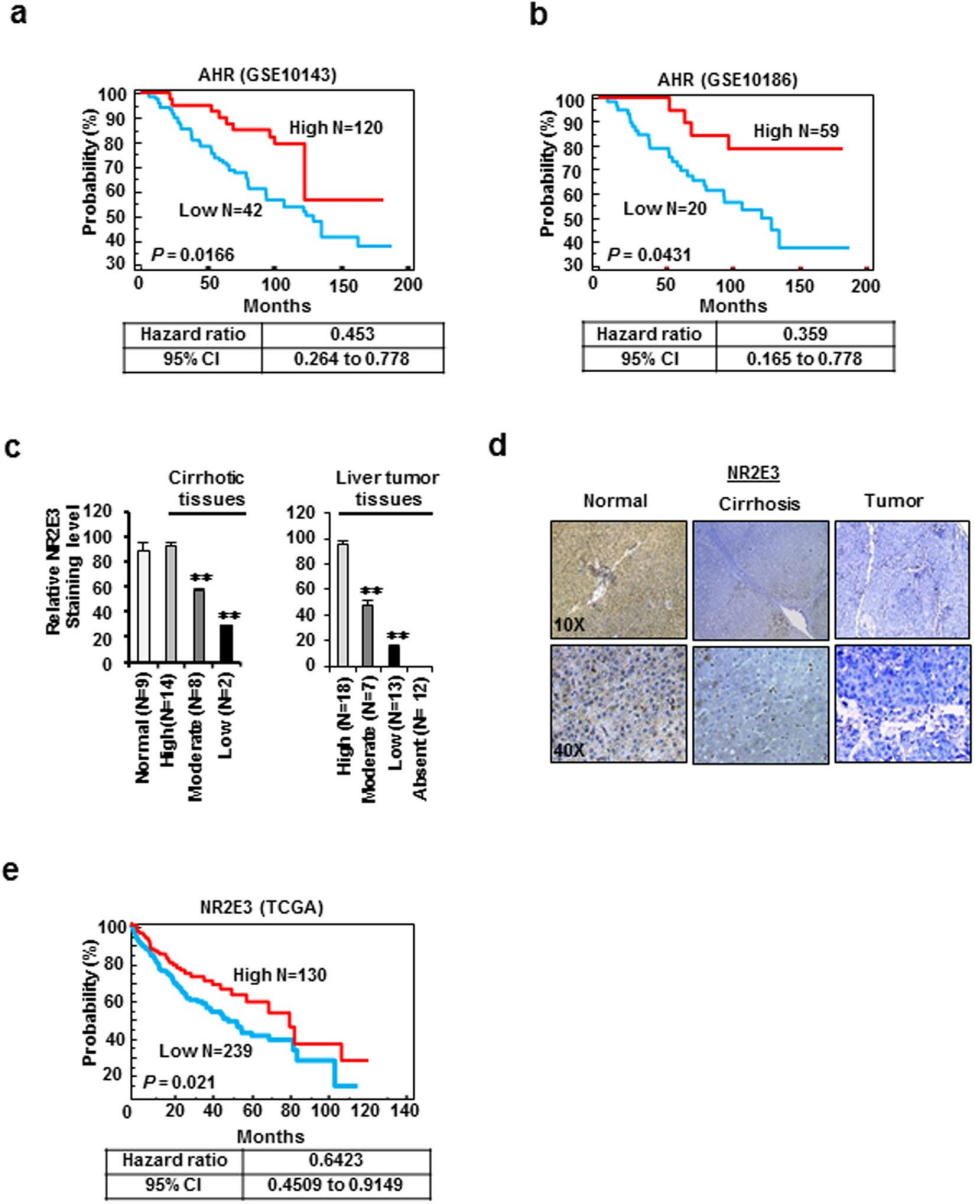
Association of *AHR* and *NR2E3* levels with precancerous liver disease and cancer. (**a**,**b**) Kaplan-Meier plot survival analysis using two independent liver cancer patient data (GSE10143, N = 162 [left]; GSE10186, N = 89 [right]). Liver cancer patients were dichotomized by relative expression levels of AHR (high vs. low). A significant P value (P < 0.05) was determined by log-rank test. The hazard ratio and 95% confidence interval for each dataset are indicated at the bottom. (**c**) Quantification of the NR2E3 st aining intensity in normal liver (N = 9), cirrhotic liver (N = 24), and liver tumor (N = 50) tissues. The NR2E3 staining intensity was classified as high (>70%), moderate (between 70 and 30%), low (<30%) and non-detectable. (**d**) Immunohistochemical staining images of NR2E3 in human cirrhotic and liver tumor tissue using tissue microarray. (**e**) Overall survival (OS) analysis based on the relative expression levels of NR2E3 mRNAs in liver cancer patients using publically available The Cancer Genome Atlas (TCGA) data (N = 369).

A previous study reported that the *Ahr* expression was decreased during liver carcinogenesis induced by diethylnitrosamine (DEN) in mice and that *Ahr* knockout mice exhibited enhanced development of DEN-induced liver tumor formation^15^. This finding indicates that *Ahr* may function as a tumor suppressor gene in liver cancer. However, the clinical association of AHR with human liver cancer has not yet been firmly established. Thus, we examined whether AHR levels are significantly correlated with prognosis in human liver cancer development. For this analysis, we employed a publicly available database (GEO in the National Center for Biotechnology Information) to retrieve gene expression data of liver cancer patients. The results of the Kaplan-Meir survival analysis using two different clinical datasets (GEO10143 and GEO10186)^46, 47^ showed that the overall survival (OS) was significantly better in patients with higher AHR expression than in patients with lower AHR expression (Fig. 6a and b, top), indicating that patients expressing high levels of AHR had good clinical outcomes. Correspondingly, the hazard ratios from each analysis result were also low (Fig. 6a and b, bottom).

We next investigated whether NR2E3 levels are altered in precancerous liver diseases such as cirrhosis and liver tumor by comparing the protein levels between diseased and normal liver tissues. Immunohistochemical staining of NR2E3 was carried out using tissue microarray analysis of human liver tissue. The NR2E3 expression levels were low in cirrhotic tissue and even lower in liver tumor tissue (Fig. 6c). Figure 6d and Supplementary Fig. 6 contain representative images showing the immunohistochemical staining of NR2E3 in normal, cirrhotic, and liver tumor tissues (Fig. 6d and Supplementary Fig. 8). Of the 24 cirrhotic liver tissues, 10 showed moderate (33%, N = 14) and low (8%, N = 2) levels of NR2E3 staining (Fig. 6, left). Furthermore, liver tumor tissue samples that were immunostained with NR2E3 antibody exhibited high (36%), moderate (14%, N = 7) low (26%) and non-detectable (24%) staining levels (Fig. 6 right). Supplementary Tables 6–8 present the clinical information of the tissue samples analyzed (Supplementary Tables 6–8). Consistently, the results obtained from a publically available database of cancer expression profiles (ww.oncomine.org) showed decreased NR2E3 mRNA expression in human cirrhotic and hepatocellular carcinoma relative to normal liver tissues (Supplementary Fig. 9).

By further employing the other independent human liver tumor gene expression data from The Cancer Genome Atlas (TCGA) which contains patient clinical information, we determined the clinical significance of NR2E3 in terms of the OS of liver cancer patients by performing Kaplan-Meier survival analysis. The results consistently indicated that higher NR2E3 levels in the patients were significantly associated with good clinical outcomes (Fig. 6e). Collectively, these results showed for the first time that both AHR and NR2E3 are good prognostic indicators and may function as tumor suppressor genes during the development of liver cancer.

## Discussion

AHR plays important roles in a wide range of cellular events to maintain cellular homeostasis, including cell growth, differentiation, transformation, and death^11, 12^. Thus, proper control of AHR levels in target tissues in response to various endogenous signals and xenobiotic chemical exposures is vital. However, our understanding of the underlying mechanism that regulates and maintains AHR expression remains largely unclear. Based on our initial indication that NR2E3-mediated signaling pathways were likely associated with AHR, our study is the first to discover a novel connection between NR2E3 and AHR and establish that NR2E3 is a novel upstream regulator of AHR. Our data demonstrated that NR2E3 specifically binds to the proximal promoter region of the AHR gene and thereby, acts as a basis for the formation of transcriptional activator complex. Several reports have shown that the AHR gene promoter lack TATA boxes and instead contains GC-rich sites for Sp1 transcription factor binding^38, 39^. By using the binding of Sp1 to the proximal region of the AHR gene as a positive control, we confirmed that NR2E3 forms a protein complex with Sp1 and a coactivator GRIP1, leading to the formation of a transcriptionally active NR2E3/GRIP1/Sp1 complex with RNA pol II (Fig. 3f and g). Consistently, the depletion of NR2E3 or GRIP1 using siRNA targeting NR2E3 or GRIP1 markedly decreased the AHR expression in both MCF-7 and HepG2 cells (Figs 2a,b and 3d,e), demonstrating that NR2E3 and GRIP1 are essential components for maintaining AHR expression. Correspondingly, the results of the ChIP assay indicated that depletion of NR2E3 disrupted the transcriptionally active complex consisting of NR2E3, GRIP1, and Sp1, leading to AHR repression (Fig. 3h and i). In NR2E3-depleted cells, the induction level of CYP1A1, a key downstream target of ligand-activated AHR, was also markedly reduced (Fig. 2c). Taken together, these results demonstrated that NR2E3 status is a key player for maintaining AHR expression and responsiveness.

The altered epigenetic status of the AHR gene promoter region was further analyzed *in vivo* using Rd7 mice. Rd7 mice have been primarily used to investigate its role in the development of retinal diseases but not to study other tissues and diseases^1–4, 40, 41^. Interestingly, we found out that Rd7 mice showed a partial phenotype; Nr2e3 proteins were not expressed in the retina but low in the liver of Rd7 mice (Fig. 4 and Supplementary Fig. 4C and D) and this was possibly due to tissue-specific differences in LINE-1 activity, splicing variation or low mRNA stability, etc^44, 45, 48^. Nonetheless, our results demonstrated that NR2E3 depletion *in vivo* induced low *Ahr* (Fig. 4a–c), and *Esr1* (mouse estrogen receptor α, another NR2E3 target^6, 7^) expressions in the livers of Rd7 mice, suggesting its positive regulative role in *Ahr* and *Esr1* expressions (Supplementary Fig. 2a,b, and Fig. 4). However, the expression of *Pparα*, which is a downstream target gene of AHR, remained unchanged between Rd7 and WT mouse livers^49^ (Supplementary Fig. 2c), indicating that NR2E3 likely regulates different sets of genes independent of AHR. We also determined whether NR2E3 depletion caused any liver damage in Rd7 mice, but no damage was observed (Supplementary Fig. 3e). Nr2e3 depletion in the livers of Rd7 mice altered the epigenetic status of the AHR gene promoter, which was similar to the changes detected at the cellular level. Together, these findings indicated that NR2E3 depletion decreased AHR expression by disrupting the active NR2E3/GRIP/Sp1 complex (Fig. 4d). Correspondingly, the induction levels and activities of CYP1A1 and CYP1A2 detoxification enzymes in the liver were significantly reduced by TCDD exposure in Rd7 mice relative to that in WT mice (Fig. 4f). Taken together, these results indicate that NR2E3 modulates the expression levels of AHR and ESR1, which are crucial molecular effectors that function in maintaining normal liver physiology, in response to environmental exposure and various endogenous signals.

LSD1 is an essential epigenetic regulator that can demethylate H3K4me2, which is related to active transcription status. This histone demethylation results in the repression of target genes^20, 21^. In our previous study, we demonstrated that NR2E3 depletion reduced H3K4me2 levels by redirecting LSD1 to the proximal promoter region of the ER gene, turning off ER gene transcription^7^. We observed a similar event where NR2E3 depletion induced repressive epigenetic status by facilitating recruitment of the corepressors Sin3A and LSD1 to the proximal promoter region of the AHR gene, accompanied by reduced H3K4me2 levels and disruption of active transcriptional complex formation (Fig. 3h and i). To further determine whether *Ahr* repression induced by *Nr2e3* depletion *in vivo* is dependent on the histone demethylase activity of LSD1, we administered SP2509, a chemical inhibitor of LSD1, into Rd7 mice^34, 50^. Surprisingly, this treatment fully restored *Ahr* and *Esr1* expression (Fig. 5a–c and Supplementary Fig. 4a and b), suggesting that the repression of these *NR2E3* target genes is dependent on histone demethylase activity of LSD1. It has become increasingly evident that LSD1 is highly oncogenic correlated with the progression of various human cancers. Indeed, the inhibition of LSD1 enzymatic activity impaired cancer cell growth, metastasis, and even cancer stem cell properties, as a result of which LSD1 is an attractive molecular target for treating various types of cancers^32–35^. Intriguingly, a significant decrease in the H3K4me2 level was observed during human liver cancer progression whereas substantially increased expression of LSD1 was previously reported; patients with higher expression of LSD1 or lower H3k4me2 marks exhibited poor clinical outcomes^28–32^. These data are highly indicative of the likely involvement of NR2E3 depletion or loss in the increased susceptibility of liver cancer development, which occurs partly via regulating LSD1 distribution and activity. Our data suggested that NR2E3 loss caused, which would probably promote susceptibility to and progression of liver cancer, in part, via modulating LSD1 redistribution and activity.

A previous study on *Ahr* knockout mice revealed significant increase in DEN-induced liver tumor formation in the knockout mice than in WT mice however without DEN exposure *Ahr* knockout mice did not exhibit any sign of damage or form liver tumor spontaneously^17^. This result suggested that *AHR* may exert tumor suppressive activity independent of its ligand-activated function. Interestingly, the tumor suppressive role of AHR has been reported in several cancers. AHR loss increased intestinal carcinogenesis in mice containing adenomatous polyposis coli gene mutation^18^. In the development of prostate cancer in transgenic adenocarcinoma of the mouse prostate (TRAMP) mice, *AHR* loss enhanced prostate carcinogenesis^20^. Furthermore, the good prognostic activity of AHR has been previously reported in breast and pancreatic cancers^51–53^. In addition to these, the results from our survival analysis using two independent clinical datasets clearly demonstrated that high AHR expression levels were strongly associated with good clinical outcomes in patients with liver cancer (Fig. 6a). Given that AHR can function as either tumor suppressor gene or oncogene^15, 16^, it is still possible that AHR may act selectively according to ligand type, etiology, subtype, and stage of liver cancer. We also observed that the NR2E3 proteins levels were decreased in some cirrhotic tissues and more reduced in liver tumors (Fig. 6c). Correspondingly, liver cancer patients who expressed high levels NR2E3 are significantly correlated with good prognosis (Fig. 6e). Overall, our results provide evidence that disruption of the NR2E3-LSD1-AHR signaling axis may play an important role in the development of precancerous liver diseases and liver cancer. The tumor suppressive role of NR2E3 during liver cancer development is currently under investigation.

Interestingly, several recent reports have demonstrated that loss of AHR caused development of retinal degenerative diseases similar to the retinal diseases caused by NR2E3 depletion. AHR deficiency in mice has been shown to cause age-related macular degeneration with atrophy in the retinal pigment epithelium^54, 55^, implicating potential crosstalk between the NR2E3 and AHR gene networks, although this possibility needs more investigation.

In summary, our work revealed the novel role of NR2E3 as a positive upstream transcriptional regulator of AHR. Loss of NR2E3 caused repression of AHR by epigenetic reprogramming, which altered the active H3K4me2 status by modulating LSD1 distribution and activity. Furthermore, our results revealed that the NR2E3 and AHR levels are significantly associated with good clinical outcomes in terms of survival in patients with liver cancer and illustrated that Rd7 mice could be used as a novel animal model for investigating the underlying molecular mechanisms associated with development of precancerous liver diseases and liver cancer. Furthermore, our results strongly suggested that decrease or loss of NR2E3 could trigger epigenetic reprogramming promoting susceptibility to and development of liver cancer such that inhibition of LSD1 or modulation of NR2E3 function can be a novel prevention or therapeutic strategy in liver cancer development, in part, by regulating expression of tumor suppressive genes. Further studies are required to examine these possibilities in different experimental settings.

## Methods

### Cell lines, reagents, plasmids, and tissue microarrays

HepG2 and MCF-7 cells were purchased from the American Type Culture Collection (ATCC, Manassas, VA) and were not further tested or authenticated by the authors. These cell lines were maintained at 37 °C in the presence of 5% CO_2_ in minimal essential medium-α (MEMα) or RPMI medium supplemented with 10% fetal bovine serum (FBS) and 1% penicillin/streptomycin solution (Sigma Aldrich, St Louis). The cells were seeded at a concentration of 2.5 × 10^4^ per well, and the FBS content of the medium was reduced to 5%. Antibodies against β-actin, ER alpha (Cat #: sc-543), AHR (cat#: sc-5579), SRC2 (Cat#: sc-135931), and RNA pol II (Cat#: sc-9001) were purchased from Santa Cruz Biotechnology (Santa Cruz, CA). NR2E3 antibodies were obtained from Aviva Systems Biology (Cat#: ARP39069 and ARP39070), Santa Cruz Biotechnology (Cat #: sc-374513 and sc-292264), Proteintech (Cat#: 14246-1-AP) and Abcam (Cat #: ab172542). Sin3A (Cat #: 8056) was purchased from Cell Signaling (Danvers, MA). H4Ac (Cat #: 39925), Sp1 (Cat #: 39058), LSD1 (Cat #: 39186) and H3K4me2 (Cat #: 39679) were procured from Active Motif (Carlsbad, CA). 2, 3, 7, 8-Tetrachlorodibenzo-*p*-dioxin (TCDD) was dissolved in DMSO, and the stock solutions were directly added to the culture media. Control cells were treated with DMSO alone. Small interfering RNAs (siRNAs) targeting GRIP1 (siRNA ID: ASI_Hs02_00341797 and SASI_Hs01_00110929) and negative control siRNAs were purchased from Sigma Aldrich. Small hairpin RNAs targeting NR2E3 were previously described^6, 7^. SP2509 was purchased from Cayman Chemical (Ann Arbor, MI). Tissue microarrays for NR2E3 staining of liver tumor and cirrhotic tissues (NBP2-30221 and NBP2-30276) were purchased from Novus Biologicals, LLC (Littleton, CO). For Kaplan-Meier survival analysis of NR2E3, liver cancer tissue array (Hliv-HCC180Sur-03) was obtained from US Biomax, Inc. (Rockville, MD). GAL4 DNA-binding domain constructs linked to various coactivators, including GAL4-SRC1, GAL4-SRC2 (Grip1), GAL4-SRC3, GAL4-PGC-1α and GAL4-Drip205 (Trap220) and a reporter luciferase construct linked to consensus dioxin response element (DRE) were previously used and verified^56^. NR2E3 expression plasmid (pcDNA4-NR2E3) was also used previously^6, 7^.

### Animals and treatment

C57BL/6 J, wild-type (WT), and homozygous Nr2e3rd7 (Rd7) mice (6-8 weeks old) were obtained from Jackson Laboratory (Bar Harbor, ME). All animal experiments were carried out in compliance with the guidelines established by the Animal Care Committee of University of Cincinnati. The animals were acclimated to temperature- and humidity-controlled rooms with a 12-h light/ dark cycle for 1 week prior to use. The mice had access to laboratory chow and tap water ad libitum. The mice were allocated for treatment with olive oil (n = 4) or TCDD (1 and 10 µg/kg; n = 4) dissolved in olive oil. For TCDD treatment, mice were intraperitoneally (i.p.) injected with 1 or 10 µg/kg TCDD dissolved in olive oil (2%, v/v). Twenty-four hours after administration, the animals were anesthetized with CO2 and liver tissues were obtained and frozen immediately in liquid nitrogen. Then, the mice were injected i.p. with the LSD1 inhibitor SP2509 (25 mg/kg) twice at 24-h intervals and then after 48 h from the last injection. Thereafter, the mice were sacrificed and their liver tissues were collected. All the animal experiments were approved by the Institutional Animal Care and Use Committee (IACUC) and College of Medicine in University of Cincinnati. All the experiments were performed in accordance with the relevant guide lines and regulations of the institutions.

### Western blotting

Either whole cell lysates or minced liver tissues homogenized in lysis buffer were collected, and equal amounts of total cellular protein (50–80 μg per lane) were resolved by sodium dodecyl sulfate-polyacrylamide gel electrophoresis (SDS-PAGE) and transferred onto polyvinylidene difluoride (PVDF) membranes. After blocking, the membranes were incubated with primary antibodies against AHR, ER, GRIP1, Sp1, or NR2E3 (1: 1000 dilutions). Proteins of interest were detected with horseradish peroxidase (HRP)-conjugated secondary antibodies (1: 3000 dilutions), and visualized with enhanced chemiluminescence (ECL) detection system using C-DiGit Blot Scanner from LI-COR (Lincoln, NE). For NR2E3 detection, NR2E3 antibodies from Aviva Systems Biology (Cat#: ARP39069 and ARP39070), Santa Cruz Biotechnology (Cat #: sc-374513), Proteintech (Cat#: 14246-1-AP) and Abcam (Cat #: ab172542) were used. β-Actin was used as the loading control.

### Chromatin immunoprecipitation assay

A chromatin immunoprecipitation (ChIP) assay was carried out using the ChiP-IT Express Enzymatic Magnetic Chromatin Immunoprecipitation Kit or the Re-ChIP-IT kit (Active Motif, Carlsbad, CA) according to the manufacturer’s protocol. Approximately 300 mg of liver tissue was minced, homogenized, and fixed with 1% formaldehyde for the *in vivo* ChIP assay. The following ChIP primer sets were used for the ChIP assay: human AHR ChIP primer set I (−283 to 90, forward): 5′-TTA GCT GAC CCA CCG TCT CT-3′, (−283 to −90, reverse): 5′-TCC ATT CCG TCT TCC TTG AG-3′; human AHR ChIP primer set II (−1969 to −1779, forward): 5′-TTA GCT GAC CCA CCG TCT CT-3′, (−1969 to −1779, reverse): 5′-TTG GCT ATT TGG TGC AGT CA-3′; Mouse AHR ChIP primer set (−138 to +141, forward): 5′-AGA ACC TCG GAC TGC AAG AA-3′, (−138∼ +141, reverse): 5′-AGT CCG TCC ACC AGT TCG T-3′. For ChIP assays, NR2E3 antibody from Santa Cruz Biotech (Cat#: sc-292264) or Aviva Systems Biology (Cat#: ARP39069) was employed. All the ChIP-PCR reactions were performed using a 7300HT Real-Time PCR System with a 96-well block module (Applied Biosystems). The cycling conditions were 56 °C for 30 min and 95 °C for 10 min, followed by 50 cycles of 95 °C for 25 s and 60 °C for 60 s.

### Real-time quantitative RT-qPCR

Total RNA was extracted from cells or liver tissue, and the cDNAs were amplified for quantitative real-time PCR, which was performed as previously described^6, 7^. The obtained data were normalized to *GAPDH* or *β-actin*. The primer set sequences used: human *AHR* (forward): 5′-CTT CCA AGC GGC ATA GAG AC-3′, (reverse): 5′-AGT TAT CCT GGC CTC CGT TT-3′; human *NR2E3* (forward): 5′-AGC AGC GGG AAG CAC TAT G-3′, (reverse) 5′-CCT GGC ACC TGT AGA TGA GC-3′; human SRC2 (Grip1) (forward): 5′-TGG GGC CTA TGA TGC TTG AG-3′, (reverse): 5′-GGT TTT TGA CAA ATT CCG TGT GG-3′; mouse *AHR* (forward): 5′-AGC CGG TGC AGA AAA CAG TAA-3′, (reverse) 5′-AGG CGG TCT AAC TCT GTG TTC-3′; mouse *Nr2e3* (forward): 5′-CCG GCT GAA GAA GTG CTT AC-3′, (reverse): 5′-TAA GGC TGG CCA TAA AGT GG-3′; mouse *Nr2e3* Exon6-8 I (forward) 5′-GCAGTGGATCCCACAGAGTT-3′, (reverse) 5′-GAGCAATTTCCCAAACCTCA-3′; mouse *Nr2e3* Exon 6-8 II (forward) GCTAAGCCAGCATAGCAAGG-3′, (reverse) 5′-CTCCATCGGAGTGTTCCCTA-3′; mouse *Esr1*(ER; forward): 5′-CCT CCC GCC TTC TAC AGG T-3′, (reverse): 5′-CAC ACG GCA CAG TAG CGA G-3′.; mouse *Ppara (*forward): 5′-AGA GCC CCA TCT GTC CTC TC-3′, (reverse)5′-ACT GGT AGT CTG CAA AAC CAA A-3′; mouse G*apdh* (forward): 5′-GCA CAG TCA AGG CCG AGA AT-3′ (reverse): 5′-GCC TTC TCC ATG GTG GTG AA-3′; mouse β-actin (forward): 5′-TGACAGGATGCAGAAGGAGA-3′; (reverse): 5′-CTG GAA GGT GGA CAG TGA GG-3′.

### Co-immunoprecipitation (Co-IP) assay

Co-immunoprecipitation assays were performed using a Universal Magnetic Co-IP Kit (Active Motif, Carlsbad, CA) according to the manufacturer’s protocol. Nr2e3 antibodies from Santa Cruz Biotech (Cat #: sc-292264 and sc-374513) were used for these co-IP assays. Briefly, MCF-7 or HepG2 cells were lysed in co-immunoprecipitation buffer containing protease inhibitor cocktail, 1 M DTT, 5 M NaCl and Igepal CA 630). The lysates were centrifuged for 10 min at 12, 000 rpm at 4 °C and the resulting supernatant that contains ~2 mg of total protein were incubated either with 3 ug of IgG or of primary antibody at 4 °C overnight in the Co-IP buffer. Then, 20 μL of protein G magnetic beads was added to the mixture and the mixture was then incubated at 4 °C for 3 h. The immunoprecipitated protein complexes were washed thrice with Co-IP buffer. After the supernatant was discarded, the antibody/protein complexes were resuspended in 40 μL of loading buffer and boiled for 5 min. The entire sample was separated with SDS-PAGE and assayed by immunoblotting to detect target protein co-immunoprecipitation.

### Immunofluorescence

Cells (5 × 10^3^ cells per chamber) were seeded into chamber culture slides (BD Falcon, Franklin Lakes, NJ). The next day, the cells were fixed with 4% paraformaldehyde for 20 min at 4 °C followed by permeabilization using ice-cold phosphate-buffered saline (PBS) containing 1% Triton X-100 and 0.5% NP-40 for 1 h. The cells were blocked with 1% BSA in PBS for 30 min on the blotter and subjected to immunofluorescence staining by incubation with primary anti-NR2E3 antibodies (Santa Cruz Biotech, Cat #: sc-292264 and sc-374513) were used (1:100 dilution) in 1% bovine serum albumin (BSA) in PBS in a humidified chamber at 4 °C overnight, followed by incubation with Alexa Fluor 594 (1:1000 dilution; Thermo Fischer Scientific) in 1% BSA for 3 h at room temperature. After washing, the second staining was carried out using anti-Grip1 or anti-Sp1 primary antibodies and Alexa Fluor 488 (1:1000 dilution) was consecutively done or vice versa. The cells were finally mounted with Prolong antifade reagent with 4′-6 diamino-2 phenylindole (DAPI; Life Technologies). The images were acquired using a Zeiss Axiovert 200 M Fluorescence/Live cell Imaging Microscope with AxiocamMR3.

### Luciferase assay

For one hybrid assay, GAL4 DNA-binding domain constructs linked to various coactivators, including GAL4-SRC1, GAL4-SRC2 (Grip1), GAL4-SRC3, GAL4-PGC-1α and GAL4-Drip205 (Trap220), were used^56^. Briefly, cells were transfected using Lipofectamine 2000 (Invitrogen) with the appropriate GAL4 plasmid and GAL4 reporter luciferase. The next day, cells were lysed and luciferase assays were performed using a Dual-Luciferase® Reporter Assay System (Promega, Madison, WI). The luciferase activity was normalized to that of renilla luciferase activity as previously described^9^. For measuring effects of NR2E3 expression on the transcriptional activation function of AHR, a reporter luciferase construct linked to consensus dioxin response element (DRE) was employed^56^. Cells were co-transfected either with equal amount of empty plasmid pcDNA4.0 (empty) as negative control or NR2E3 expression plasmid (pcDNA4-NR2E3)^6, 7^ and DRE reporter luciferase, and cells were treated with TCDD on the next day. After 16 h, the cells were lysed and luciferase assays were performed as previously descirbed^6, 7, 56^.

### Ethoxyresorufin-*O*-deethylase (EROD) and alanine aminotrasferase assays

Ethoxyresorufin *O*-deethylase (EROD) activities were measured using different subcellular fractions from TCDD-treated and control liver tissues from both WT and RD7 mice. The reaction mixture contains 25 μg protein with 0.1 M NADPH, 7-ethoxyresorufin (approximately 0.1 mg/mL) and Tris-NaCl (TN) buffer (0.05 M Tris, pH 7.6, containing 0.1 M NaCl). Each reaction was performed as previously described^57^. The alanine aminotrasferase (ALT) assay was performed according to the manufacturer’s protocol (BioVision, Inc., Milpitas, CA).

### Histone extraction and quantification of the H3K4me2 histone modification level

For measuring the global H3K4 di-methylation levels, the EpiQuik™ Global Di-Methyl Histone H3-K4 Quantification Kit (EpiGenTek, Farmingdale, NY) was employed. The histone extraction and colorimetric assay for measuring global H3K4me2 levels were both carried out according to the manufacturer’s protocol.

### Immunohistochemical staining

After the sections were deparaffinized in xylene and rehydrated through a series of graded ethanol solutions, antigenic retrieval was performed by immersing the sections in 0.01 mM sodium citrate (pH 6.0) and heating in an microwave oven (100 °C) for 9 min. Deparaffinized sections were incubated with peroxidase-blocking reagent (Biogenex, CA, USA) for 9 min in a humidified chamber to block endogenous peroxidase activity. After blocking nonspecific binding sites with nonspecific staining blocking reagent (Vector Laboratories, Burlingame, CA, USA) for 30 min, the sections were incubated with primary antibodies at 4 °C overnight. Subsequently, peroxidase-conjugated secondary antibodies (Vector Laboratories) and 3,3-diaminobenzine-tetrachloride (DAB; Vector Laboratories) were used according to the manufacturer’s instructions. The sections were counterstained with hematoxylin and observed under a microscope.

### Differential gene expression

Differential gene expression between NR2E3 knockout and control MCF7 cells: Bioconductor/R limma package^58^ was used to predict differentially expressed genes (DEGs) between the NR2E3 knockout (n = 3) and control (n = 3) groups from the previously published MCF7 dataset (GSE18431). Genes were considered differentially expressed when they passed the cutoff criterion of p < 0.0005.

Differential gene expression between NR2E3 knockout and control HepG2 cells**:** To identify the set of DEGs between NR2E3 knockout Hep2G cells (n = 2) and control HepG2 cells (n = 2), we used single-end RNA-seq data. These RNA-seq data were deposited in the Gene Expression Omnibus (GEO) website (GSE79463). Briefly, single-end reads were aligned to the mm10 genome from UCSC (downloaded from Illumina’s iGenomes repository, https://support.illumina.com/sequencing/sequencing_software/ igenome.html) using TopHat^59^, then read counts were computed using FeatureCounts^60^. Differential gene expression analyses were carried out using the DESeq. 2 method^61^. Genes were considered differentially expressed when they passed the cutoff criterion of p < 0.0005. These RNA-seq data were deposited in the Gene Expression Omnibus (GEO) website (GSE79463).

### Common gene sets

A total of 252 common genes were collected by intersecting two DEG sets described above (microarray and RNA-seq). Heatmaps were generated using the R/pheatmap package. The log2 expression values of these common genes were subtracted by the average log2-expression values of the control to visualize log2-fold changes in heatmaps. Genes were clustered using the hierarchical clustering method. Figure 1b presents two clusters, including AHR.

### Gene set enrichment analysis

We used WebGestalt41 for gene set enrichment analysis for 252 common genes^62^. Differentially expressed gene sets were queried against the WikiPathway gene sets and an FDR cutoff of <0.05 was applied to select significantly enriched gene sets/pathways. Figure 1c presents the gene sets associated with AHR signaling.

### Pre-ranked GSEA analysis

GSEA analysis^63^ was performed using a pre-ranked option (all genes) because conventional GSEA is not optimized for RNA-seq data. The ranked list was generated according to the inverse of P values with +/− (i.e. up/down) signs and classic weighting method was applied^64^. Gene sets defined in the KEGG database were used for reference.

### Determination of changes in the NR2E3 mRNA level and prognostic activity using human clinical liver datasets

We mined data using Oncomine (https://www.oncomine.org/). Two datasets (GSE14520 and GSE6764) were used for the analysis (Supplementary Figure 6)^36, 37, 65^. The Kaplan-Meir survival analysis was performed using two clinical datasets (GSE10143 and GSE10186)^46, 47^. The TCGA RNAseq and clinical data of 369 liver cancer cases were downloaded from Genomic Data Commons Data Portal at National Cancer Institute (https://gdc-portal.nci.nih.gov/). Only upper quartile normalized fragments per kilobase of transcript per million mapped reads (UQ-FPKM) were extracted for survival analysis. The cohort was then stratified into two high or low expression of NR2E3 based on their expression value. Overall survival was determined by days to last follow up and days to death.

### Statistical analysis

Results are representative of at least 2-3 independent experiments with three replicates per experiment. A one-way analysis of variance (ANOVA) was used to determine the significance of the differences between treatment groups. The Newman–Keuls test was used for multi-group comparisons. Statistical significance was set at a P value of <0.05.

## Electronic supplementary material

Supplementary Info
