# Supplementary material for: Loss of NR2E3 represses AHR by LSD1 reprogramming, is associated with poor prognosis in liver cancer
Source: Sci Rep. 2017 Sep 6;7:10662. doi: 10.1038/s41598-017-11106-2 (PMC5587550; doi:10.1038/s41598-017-11106-2)
Supplement: Supplementary file 1 — Supplementary Info [file 41598_2017_11106_MOESM1_ESM.pdf]

# Supplementary Information

## Loss of NR2E3 represses AHR by LSD1 reprogramming, is associated with poor prognosis in liver cancer

Tilak Khanal [1], Kwangmin Choi[2], Yuet-Kin Leung[1,4,5], Jiang Wang[3], Dasom Kim[1], Vinothini Janakiram[1,4], Sung-Gook Cho[4], Alvaro Puga[1,4], Shuk-Mei Ho[1,4,5,7] and Kyounghyun Kim[1,4*]

[1] Department of Environmental Health, University of Cincinnati, College of Medicine, 160 Panzeca way., Cincinnati, OH 45267

[2] Division of Experimental Hematology and Cancer Biology, Cincinnati Children's Hospital medical Center, Cincinnati, OH 45229

[3]Department of Pathology and Laboratory Medicine, University of Cincinnati, College of Medicine, 231 Albert Sabin Way., Cincinnati, OH 45267

[4]Center for Environmental Genetics (*P30 ES006096), University of Cincinnati, College of Medicine,* 160 Panzeca way., Cincinnati, OH 45267

[5]Cincinnati Cancer Center, 231 Albert Sabin Way, Cincinnati, OH 45267

[6] Department of Biotechnology, Korea National University of Transportation, Chung-ju, South Korea.

[7]Cincinnati VA Medical Center, 3200 Vine Street, Cincinnati, OH 45220

Correspondence and requests for materials should be addressed to K.K. (email:kim2ku@uc.edu)

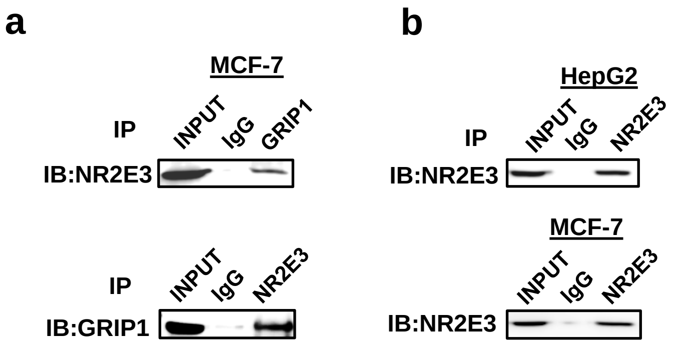


**Supplementary Figure 1. NR2E3-GRIP1 interaction in MCF-7 human breast cancer cells and evaluation of anti-NR2E3 antibodies.** (a) Co-Immunoprecipitation (IP) assay was performed to confirm NR2E3-GRIP1 interaction in MCF-7 cells. Anti-NR2E3 antibody (sc-374513), anti-Grip1 antibody or control non-immune IgG (IgG) was used for immunoprecipitation and immunoblot analysis (IB) was performed detecting GRIP1 (Top) or vice versa (Bottom). (b) To validate whether anit-NR2E3 antibody efficiently precipitate NR2E3 protein, Co-IP assays was carried out. A rabbit polyclonal anti-NR2E3 (Santa Cruz Biotech, Cat#: sc-292264) was used for pulling down NR2E3 protein either from HepG2 (Top) or MCF-7 whole cell lysate (Bottom) and then NR2E3 was detected by immunoblotting with a mouse monoclonal NR2E3 antibody (Santa Cruz Biotech, Cat #: sc-374513). Images of uncropped western blots and gels can be found in Supplementary Fig. 11.

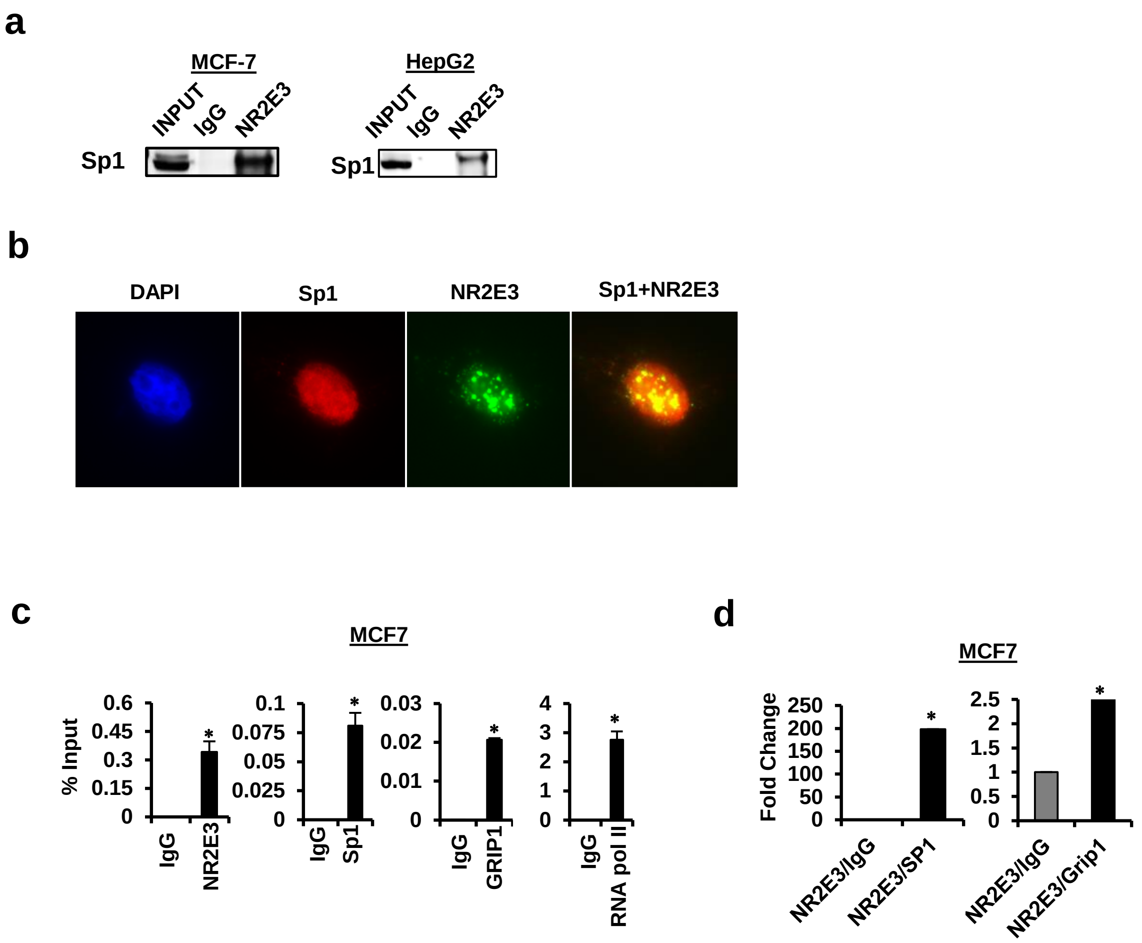


**Supplementary Figure 2. The formation of NR2E3-mediated transcriptionally active complex with Sp1 and GRIP1 on the AHR gene promoter in MCF-7 cells.** (a) Sp1 was co-immunoprecipitaed with NR2E3 using MCF-7 cell lysate. (b) The nuclear co-localization of NR2E3 (green) with Sp1 (red) in HepG2 cells as shown as yellow speckles. (c) Binding of NR2E3, Sp1, GRIP1 and RNA pol II to the proximal promoter region of AHR gene in MCF7 cells as determined by ChIP-PCR. (d) Re-ChIP PCR results indicates the formation of NR2E3/Sp1 and NR2E3/ GRIP1 protein complex in the AHR proximal promoter region in MCF7 cells. Results are means ± SE for at least 3 replicated determinations, and significantly ($P < .05$) increased (*) responses are indicated. Images of uncropped western blots and gels can be found in Supplementary Fig. 11.

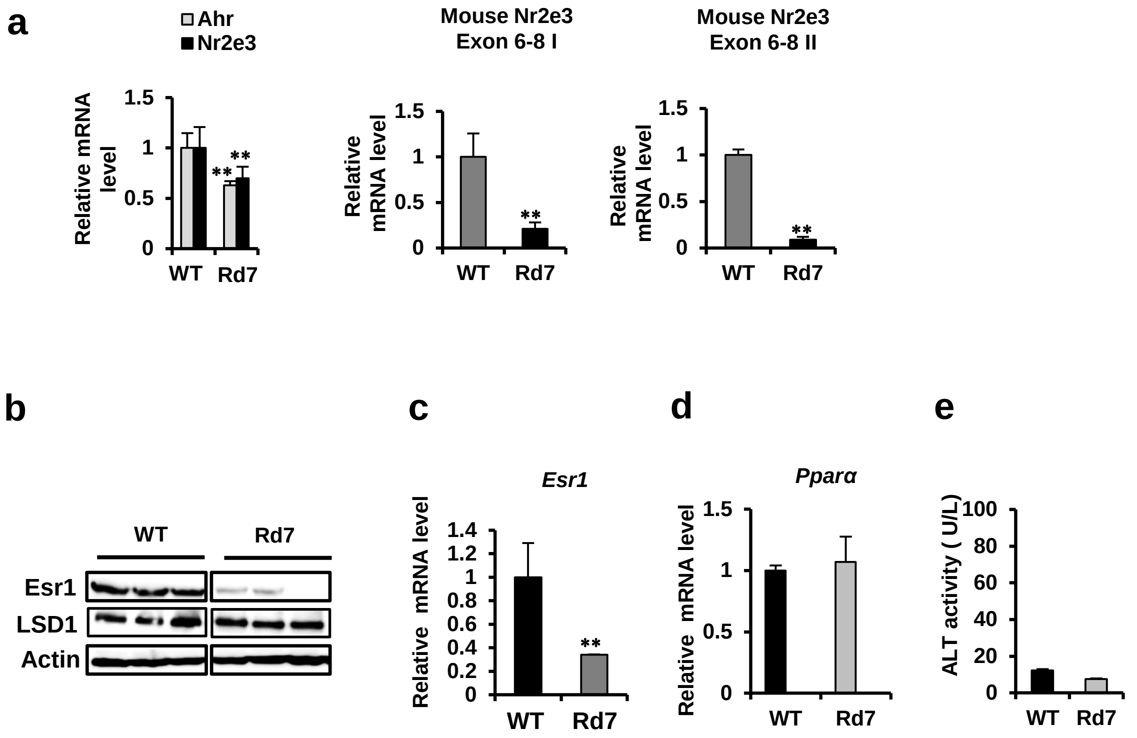


**Supplementary Figure 3. Effects of NR2E3 depletion on the level of Nr2e3, Esr1 and other Ahr target gene expressions and liver damage *in vivo*.** (a) Changes in mRNA levels of Nr2e3 and Ahr in Rd7 mice was determined by qRT-PCR analysis using the primer set amplifying Ahr and three primer sets amplifying different regions of Nr2e3 gene [Nr2e3, *Nr2e3* Exon6-8 I and *Nr2e3* Exon 6-8 II]. (b) The livers were homogenized, and the ER protein levels were analyzed on immunoblots of cell lysates probed with anti-ER and anti-LSD1 antibodies. (c-d) The livers were homogenized and lysed, and the total RNA was prepared for the PCR analysis of Esr1 and Pparα mRNA expression relative to the GAPDH expression. (d) Measurement of alanine aminotrasnfersae (ALT) activity, a indicator of liver damage, using serum from wild type and Rd7 mice. Results are means ± SE for at least 3 replicated determinations, and significantly (P < .05) decreased (**) response is indicated. Images of uncropped western blots and gels can be found in Supplementary Fig. 11.

Anti-NR2E3 antibody (Abcam, Cat #172542)

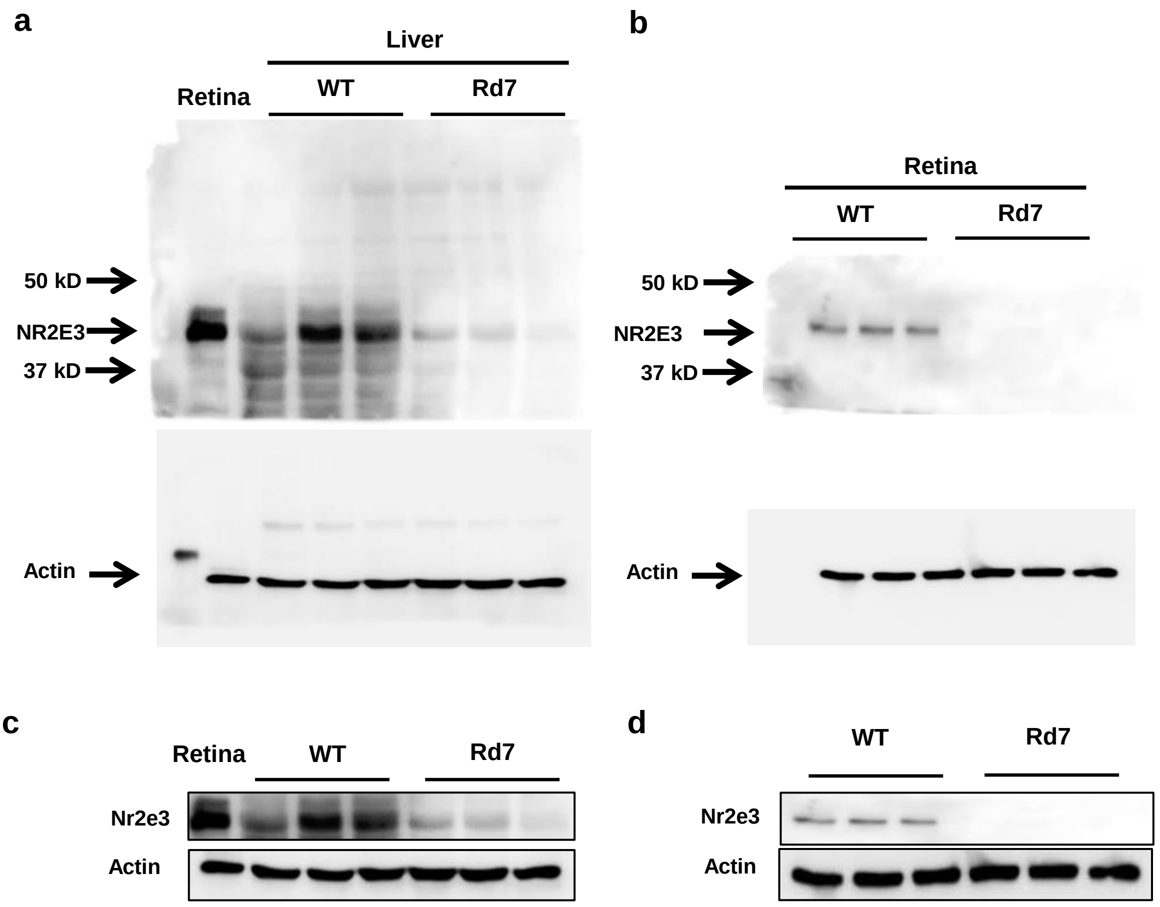


**Supplementary Figure 4. *Nr2e3* protein expression in the liver and retina of Rd7 mice.** (a) The original blot image for Nr2e3 protein detection in the liver of WT and Rd7 mice using C57/BL6 mouse retina lysate (Zyagen, Inc., Cat# MT-106-C57) as positive control. (b) Nr2e3 protein levels were also detected using retina lysate extracted from WT and Rd7 mice. In each lane, 30-50 μg of total protein lysate was loaded. Nr2e3 protein band was detected with NR2E3 antibody (Abcam, Cat # ab172542). The control beta-actin bands were shown down below (c, d). *Nr2e3* proteins were expressed low in the liver but not the retina of Rd7 mice.

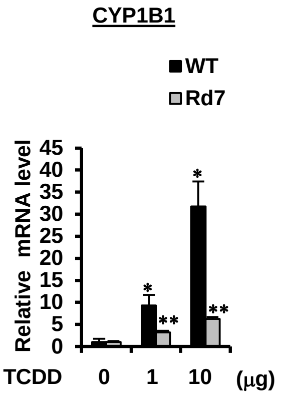


**Supplementary Figure 5. Effects of NR2E3 loss on the induction level of CYP1B1 in RD7 mice after TCDD treatment *in vivo.*** The induction levels of CYP1B1 mRNA was significantly reduced in Rd7 mice. Results are means ± SE for at least 3 replicated determinations, and significantly ($P < .05$) increased (*) or attenuated (**) responses are indicated.

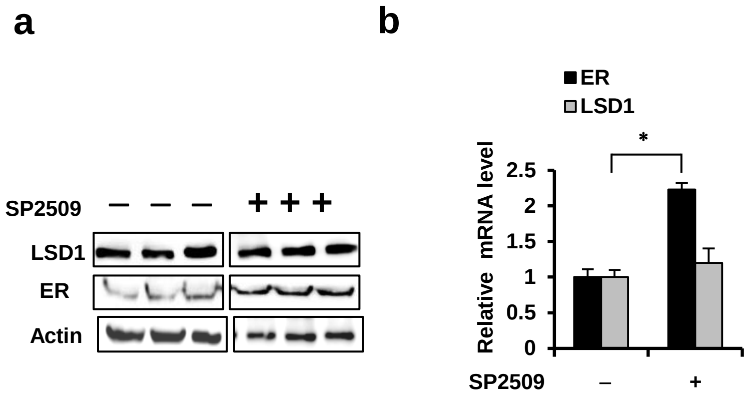


**Supplementary Figure 6. Effects of LSD1 inhibitor treatment on AHR expression.** (a) The livers were homogenized, and the LSD1 and ER protein levels were analyzed on immunoblots of cell lysates probed with anti-LSD1 and anti-ER antibodies. (b) The livers were homogenized and lysed, and the total RNA was prepared for the PCR analysis of LSD1 and ER mRNA expression relative to the GAPDH expression. Results are means ± SE for at least 3 replicated determinations, and significantly ($P < .05$) increased (*) response is indicated. Images of uncropped western blots and gels can be found in Supplementary Fig. 11.

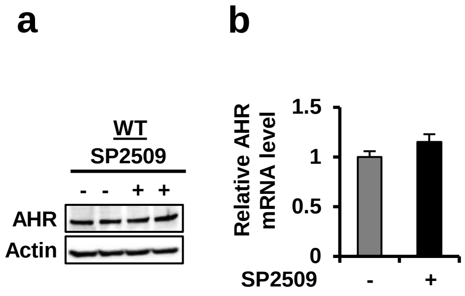


**Supplementary Figure 7. Effects of LSD1 inhibitor treatment on AHR expression in wild type mice.** (a) Wild type mouse livers treated with and without SP2509 were homogenized, and the AHR protein levels were analyzed on immunoblots of cell lysates probed with anti-AHR antibodies. (b) The livers were homogenized and lysed, and the total RNA was prepared for the PCR analysis of NR2E3 and AHR mRNA expression relative to the GAPDH expression. Images of uncropped western blots and gels can be found in Supplementary Fig. 11.

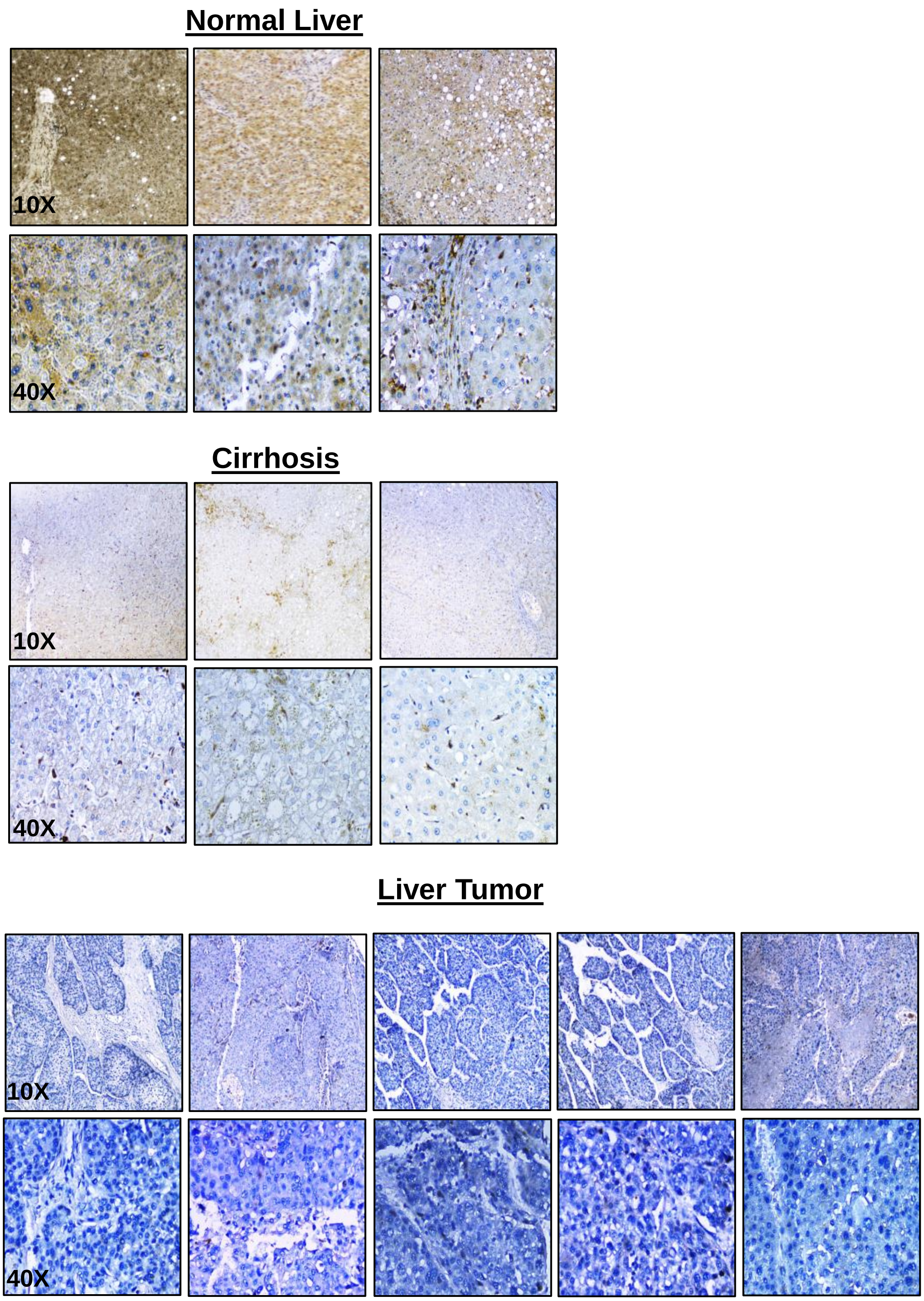


**Supplementary Figure 8**. Representative Images for immunostaining of NR2E3 in normal, cirrhotic and tumor tissues (10X and 40X magnification).

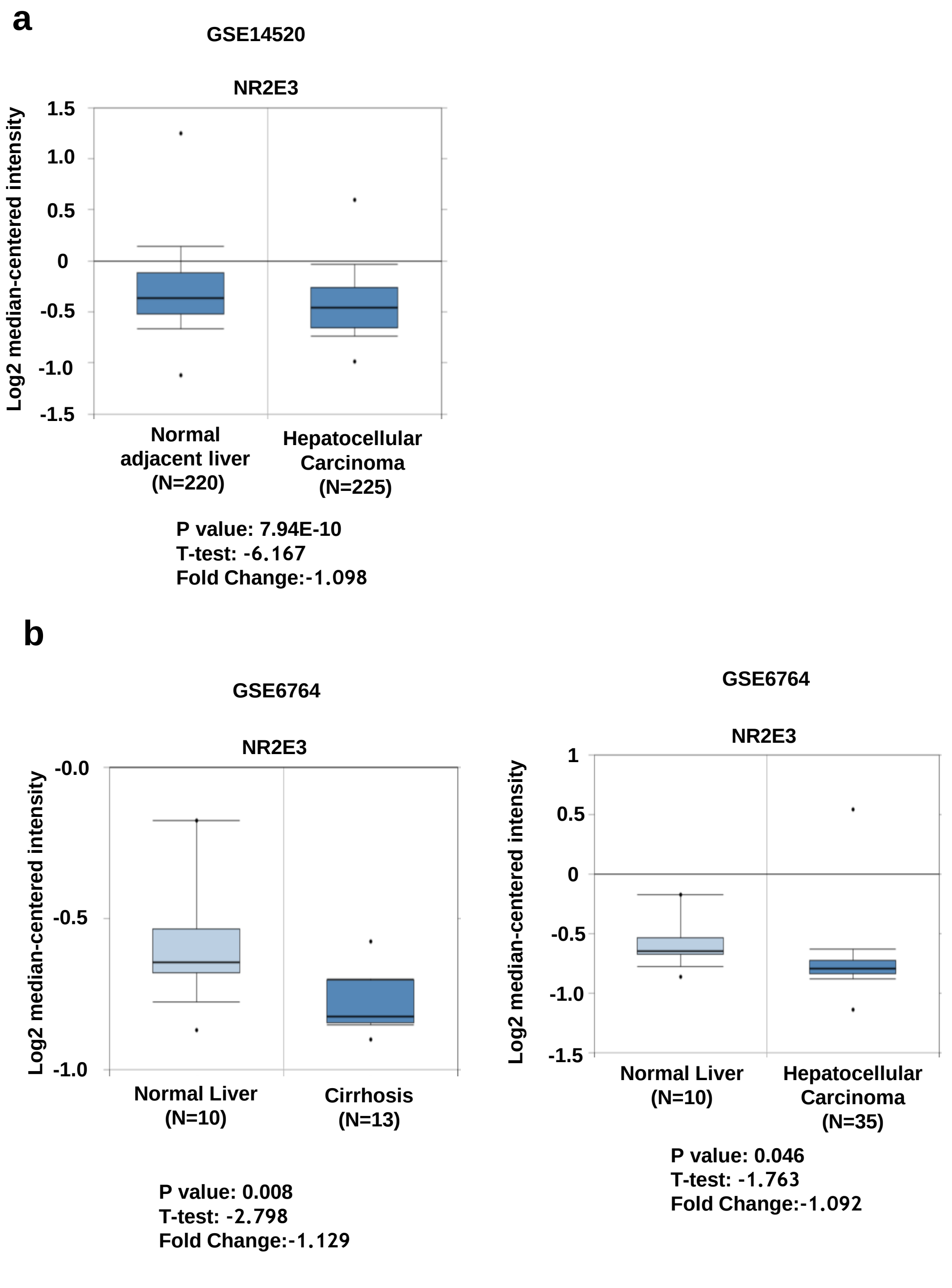


**Supplementary Figure 9**. Expression levels of NR2E3 mRNAs in normal, cirrhotic and hepatocelluar carcinoma in two independent clinical GEO data sets . (a) NR2E3 levels were decreased in human hepatocelluar carcinoma patient samples (GEO1452). (b) Reduced NR2E3 levels s were detected in cirrhotic (b, left) and hepatocelluarcarcinoma  (b, right) (GSE6764).

**Supplementary Table 1.** Major signaling pathways that are significantly associated with down-regulated gene signature induced by NR2E3 deletion in HepG2 cells.

| NAME | SIZE | ES | NES | NOM p-val | FDR q-val | FWER p-val |
|---|---|---|---|---|---|---|
| KEGG_DNA_REPLICATION | 36 | -0.587 | -4.185 | 0.000 | 0.000 | 0.000 |
| KEGG_CELL_CYCLE | 121 | -0.318 | -4.040 | 0.000 | 0.000 | 0.000 |
| KEGG_COMPLEMENT_AND_COAGULATION_CASCADES | 60 | -0.403 | -3.773 | 0.000 | 0.000 | 0.000 |
| KEGG_NUCLEOTIDE_EXCISION_REPAIR | 44 | -0.412 | -3.295 | 0.000 | 0.000 | 0.000 |
| KEGG_STARCH_AND_SUCROSE_METABOLISM | 45 | -0.403 | -3.239 | 0.000 | 0.000 | 0.000 |
| KEGG_HOMOLOGOUS_RECOMBINATION | 28 | -0.504 | -3.162 | 0.000 | 0.000 | 0.000 |
| **KEGG_DRUG_METABOLISM_CYTOCHROME_P450** | 55 | -0.359 | -3.107 | 0.000 | 0.000 | 0.000 |
| KEGG_PROPANOATE_METABOLISM | 30 | -0.465 | -3.050 | 0.000 | 0.000 | 0.000 |
| KEGG_HUNTINGTONS_DISEASE | 160 | -0.210 | -3.048 | 0.000 | 0.000 | 0.000 |
| KEGG_MISMATCH_REPAIR | 23 | -0.527 | -3.047 | 0.000 | 0.000 | 0.000 |
| **KEGG_METABOLISM_OF_XENOBIOTICS_BY_CYTOCHROME_P450** | 58 | -0.335 | -3.021 | 0.000 | 0.000 | 0.000 |
| KEGG_FATTY_ACID_METABOLISM | 35 | -0.424 | -2.962 | 0.000 | 0.000 | 0.000 |
| KEGG_ALANINE_ASPARTATE_AND_GLUTAMATE_METABOLISM | 29 | -0.425 | -2.686 | 0.000 | 0.000 | 0.001 |
| KEGG_ALZHEIMERS_DISEASE | 142 | -0.194 | -2.688 | 0.000 | 0.000 | 0.001 |
| KEGG_CITRATE_CYCLE_TCA_CYCLE | 29 | -0.411 | -2.715 | 0.000 | 0.000 | 0.001 |
| KEGG_LYSINE_DEGRADATION | 43 | -0.352 | -2.745 | 0.000 | 0.000 | 0.001 |
| KEGG_OOCYTE_MEIOSIS | 100 | -0.241 | -2.795 | 0.000 | 0.000 | 0.001 |
| KEGG_SPLICEOSOME | 121 | -0.218 | -2.841 | 0.000 | 0.000 | 0.001 |
| KEGG_PYRIMIDINE_METABOLISM | 94 | -0.257 | -2.847 | 0.000 | 0.000 | 0.001 |
| KEGG_STEROID_HORMONE_BIOSYNTHESIS | 45 | -0.354 | -2.856 | 0.000 | 0.000 | 0.001 |
| KEGG_N_GLYCAN_BIOSYNTHESIS | 46 | -0.356 | -2.867 | 0.000 | 0.000 | 0.001 |
| KEGG_OXIDATIVE_PHOSPHORYLATION | 108 | -0.239 | -2.876 | 0.000 | 0.000 | 0.001 |
| KEGG_VALINE_LEUCINE_AND_ISOLEUCINE_DEGRADATION | 44 | -0.340 | -2.634 | 0.000 | 0.000 | 0.003 |
| KEGG_PEROXISOME | 75 | -0.259 | -2.603 | 0.000 | 0.000 | 0.005 |
| KEGG_PENTOSE_AND_GLUCURONATE_INTERCONVERSIONS | 26 | -0.422 | -2.537 | 0.000 | 0.000 | 0.008 |
| KEGG_PARKINSONS_DISEASE | 102 | -0.217 | -2.528 | 0.000 | 0.000 | 0.009 |
| KEGG_PROGESTERONE_MEDIATED_OOCYTE_MATURATION | 75 | -0.238 | -2.440 | 0.002 | 0.001 | 0.022 |
| KEGG_SYSTEMIC_LUPUS_ERYTHEMATOSUS | 86 | -0.227 | -2.451 | 0.000 | 0.001 | 0.022 |
| KEGG_BASE_EXCISION_REPAIR | 33 | -0.349 | -2.400 | 0.000 | 0.001 | 0.028 |
| KEGG_PURINE_METABOLISM | 139 | -0.175 | -2.404 | 0.000 | 0.001 | 0.028 |
| KEGG_ASCORBATE_AND_ALDARATE_METABOLISM | 24 | -0.410 | -2.387 | 0.000 | 0.001 | 0.031 |
| KEGG_RETINOL_METABOLISM | 43 | -0.294 | -2.344 | 0.000 | 0.002 | 0.040 |
| KEGG_PPAR_SIGNALING_PATHWAY | 55 | -0.268 | -2.340 | 0.002 | 0.002 | 0.042 |
| KEGG_PROXIMAL_TUBULE_BICARBONATE_RECLAMATION | 21 | -0.421 | -2.300 | 0.000 | 0.002 | 0.056 |
| KEGG_DRUG_METABOLISM_OTHER_ENZYMES | 45 | -0.282 | -2.288 | 0.000 | 0.002 | 0.060 |
| KEGG_GLUTATHIONE_METABOLISM | 47 | -0.277 | -2.235 | 0.000 | 0.003 | 0.084 |
| KEGG_PENTOSE_PHOSPHATE_PATHWAY | 23 | -0.370 | -2.202 | 0.000 | 0.004 | 0.108 |
| KEGG_GLYCOLYSIS_GLUCONEOGENESIS | 50 | -0.262 | -2.154 | 0.004 | 0.005 | 0.145 |
| KEGG_LYSOSOME | 113 | -0.168 | -2.096 | 0.000 | 0.008 | 0.210 |
| KEGG_BIOSYNTHESIS_OF_UNSATURATED_FATTY_ACIDS | 22 | -0.378 | -2.071 | 0.000 | 0.009 | 0.245 |
| KEGG_PRIMARY_BILE_ACID_BIOSYNTHESIS | 12 | -0.481 | -2.013 | 0.002 | 0.012 | 0.335 |
| KEGG_ARGININE_AND_PROLINE_METABOLISM | 51 | -0.235 | -1.973 | 0.010 | 0.016 | 0.411 |
| KEGG_BUTANOATE_METABOLISM | 32 | -0.289 | -1.975 | 0.000 | 0.016 | 0.408 |
| KEGG_P53_SIGNALING_PATHWAY | 65 | -0.184 | -1.767 | 0.014 | 0.047 | 0.818 |
| KEGG_BETA_ALANINE_METABOLISM | 21 | -0.329 | -1.771 | 0.018 | 0.047 | 0.808 |

**Supplementary Table 2.** Major signaling pathways that are significantly associated with up-regulated gene signature induced by NR2E3 deletion in HepG2 cells.

| NAME | ES | NES | NOM p-val | FDR q-val | FWER p-val |
|---|---|---|---|---|---|
| KEGG_ENDOCYTOSIS | 0.2388 | 3.5523 | 0.0000 | 0.0000 | 0.0000 |
| KEGG_MAPK_SIGNALING_PATHWAY | 0.1770 | 3.0455 | 0.0000 | 0.0000 | 0.0000 |
| KEGG_RIG_I_LIKE_RECEPTOR_SIGNALING_PATHWAY | 0.3143 | 2.5944 | 0.0000 | 0.0028 | 0.0110 |
| KEGG_EPITHELIAL_CELL_SIGNALING_IN_HELICOBACTER_PYLORI_INFECTION | 0.2543 | 2.4216 | 0.0000 | 0.0056 | 0.0280 |
| KEGG_PATHWAYS_IN_CANCER | 0.1180 | 2.3296 | 0.0000 | 0.0083 | 0.0530 |
| KEGG_HYPERTROPHIC_CARDIOMYOPATHY_HCM | 0.2553 | 2.2701 | 0.0000 | 0.0117 | 0.0860 |
| KEGG_SMALL_CELL_LUNG_CANCER | 0.2225 | 2.2420 | 0.0000 | 0.0121 | 0.1010 |
| KEGG_JAK_STAT_SIGNALING_PATHWAY | 0.1867 | 2.2072 | 0.0020 | 0.0122 | 0.1300 |
| KEGG_CYTOKINE_CYTOKINE_RECEPTOR_INTERACTION | 0.1502 | 2.2093 | 0.0000 | 0.0134 | 0.1280 |
| KEGG_NEUROACTIVE_LIGAND_RECEPTOR_INTERACTION | 0.1621 | 2.1049 | 0.0021 | 0.0197 | 0.2210 |
| KEGG_CIRCADIAN_RHYTHM_MAMMAL | 0.4645 | 2.0350 | 0.0041 | 0.0278 | 0.3260 |
| KEGG_RIBOSOME | 0.1855 | 1.9918 | 0.0082 | 0.0325 | 0.4020 |
| KEGG_APOPTOSIS | 0.1926 | 1.9747 | 0.0060 | 0.0330 | 0.4330 |
| KEGG_REGULATION_OF_ACTIN_CYTOSKELETON | 0.1286 | 1.9559 | 0.0099 | 0.0337 | 0.4650 |
| KEGG_REGULATION_OF_AUTOPHAGY | 0.3494 | 1.8318 | 0.0157 | 0.0571 | 0.7240 |
| KEGG_NEUROTROPHIN_SIGNALING_PATHWAY | 0.1521 | 1.8404 | 0.0141 | 0.0580 | 0.7060 |
| KEGG_FOCAL_ADHESION | 0.1257 | 1.8475 | 0.0121 | 0.0597 | 0.6940 |
| KEGG_HEMATOPOIETIC_CELL_LINEAGE | 0.2074 | 1.7542 | 0.0176 | 0.0810 | 0.8500 |
| KEGG_SNARE_INTERACTIONS_IN_VESICULAR_TRANSPORT | 0.2423 | 1.7406 | 0.0237 | 0.0819 | 0.8670 |
| KEGG_TYPE_II_DIABETES_MELLITUS | 0.2457 | 1.6798 | 0.0309 | 0.1055 | 0.9310 |
| KEGG_DILATED_CARDIOMYOPATHY | 0.1705 | 1.6508 | 0.0395 | 0.1166 | 0.9600 |
| KEGG_ARRHYTHMOGENIC_RIGHT_VENTRICULAR_CARDIOMYOPATHY_ARVC | 0.1857 | 1.5860 | 0.0450 | 0.1504 | 0.9880 |
| KEGG_NATURAL_KILLER_CELL_MEDIATED_CYTOTOXICITY | 0.1414 | 1.5713 | 0.0464 | 0.1543 | 0.9920 |

**Supplementary Table 3.** Common 252 genes regulated by NR2E3 deletion in both MCF-7 and HepG2 cells.

| SYMBOL | Description | MCF-7 | | HepG2 | |
|---|---|---|---|---|---|
| | | Fold Change | P value | Fold Change | P value |
| ABLIM1 | actin binding LIM protein 1 | 2.17 | 6.77E-09 | 0.49 | 9.99E-18 |
| ACOT9 | acyl-CoA thioesterase 9 | 1.47 | 6.87E-05 | 1.83 | 8.29E-08 |
| ACTA2 | actin, alpha 2, smooth muscle, aorta | 1.85 | 2.01E-07 | 0.66 | 2.49E-05 |
| ADAR | adenosine deaminase, RNA-specific | 0.98 | 8.59E-01 | 0.71 | 3.09E-05 |
| ADCY9 | adenylate cyclase 9 | 0.71 | 3.21E-04 | 0.72 | 4.63E-04 |
| ADD3 | adducin 3 (gamma) | 0.70 | 7.09E-05 | 0.59 | 1.14E-07 |
| AHR | aryl hydrocarbon receptor | 0.61 | 1.08E-04 | 0.65 | 8.23E-06 |
| AIF1L | allograft inflammatory factor 1-like | 0.70 | 2.50E-04 | 0.28 | 2.04E-17 |
| AK4 | adenylate kinase 4 | 0.52 | 7.92E-08 | 0.44 | 3.18E-20 |
| AKR1B10 | aldo-keto reductase family 1, member B10 (aldose reductase) | 1.43 | 1.53E-04 | 0.50 | 6.60E-09 |
| AKR1D1 | aldo-keto reductase family 1, member D1 | 0.58 | 1.67E-06 | 0.26 | 1.55E-07 |
| ALDH3A2 | aldehyde dehydrogenase 3 family, member A2 | 0.63 | 6.59E-05 | 0.54 | 4.43E-12 |
| ALDH4A1 | aldehyde dehydrogenase 4 family, member A1 | 0.56 | 2.19E-07 | 0.47 | 9.29E-17 |
| ANKRD1 | ankyrin repeat domain 1 (cardiac muscle) | 1.52 | 1.24E-04 | 4.76 | 6.94E-89 |
| ANKRD13D | ankyrin repeat domain 13 family, member D | 0.70 | 1.64E-04 | 1.91 | 2.83E-04 |
| ANTXR1 | anthrax toxin receptor 1 | 0.66 | 4.22E-05 | 0.14 | 3.59E-51 |
| ANXA1 | annexin A1 | 5.27 | 1.48E-13 | 3.55 | 4.52E-14 |
| ANXA3 | annexin A3 | 1.76 | 2.12E-06 | 2.39 | 2.24E-19 |
| AP4B1 | adaptor-related protein complex 4, beta 1 subunit | 0.70 | 4.48E-04 | 3.53 | 2.77E-09 |
| ARHGDIB | Rho GDP dissociation inhibitor (GDI) beta | 1.44 | 1.56E-04 | 17.96 | 1.61E-11 |
| ASS1 | argininosuccinate synthase 1 | 1.71 | 1.71E-06 | 0.47 | 6.62E-10 |
| AXIN2 | axin 2 | 0.59 | 1.34E-06 | 0.35 | 6.22E-20 |
| B2M | beta-2-microglobulin | 1.89 | 6.48E-08 | 0.72 | 3.68E-05 |
| BCAS4 | breast carcinoma amplified sequence 4 | 1.54 | 1.31E-05 | 2.71 | 3.33E-06 |
| BCL2L13 | BCL2-like 13 (apoptosis facilitator) | 1.49 | 3.26E-05 | 1.56 | 4.11E-05 |
| BHLHE41 | basic helix-loop-helix family, member e41 | 0.59 | 1.83E-06 | 5.36 | 1.66E-13 |
| BIRC3 | baculoviral IAP repeat containing 3 | 1.80 | 3.26E-07 | 3.66 | 4.52E-23 |
| BLOC1S6 | biogenesis of lysosomal organelles complex-1, subunit 6, pallidin | 0.61 | 3.50E-06 | 0.62 | 3.49E-05 |
| BLZF1 | basic leucine zipper nuclear factor 1 | 0.66 | 2.70E-05 | 2.41 | 2.95E-04 |
| BMPR2 | bone morphogenetic protein receptor, type II (serine/threonine kinase) | 0.66 | 3.96E-05 | 0.51 | 1.58E-12 |
| BNIP3L | BCL2/adenovirus E1B 19kDa interacting protein 3-like | 0.67 | 3.65E-05 | 1.59 | 5.22E-08 |
| BRI3BP | BRI3 binding protein | 0.50 | 1.49E-08 | 0.23 | 3.49E-14 |
| BTG3 | BTG family, member 3 | 1.53 | 1.31E-05 | 2.12 | 7.82E-09 |
| BZW1 | basic leucine zipper and W2 domains 1 | 0.66 | 2.59E-04 | 1.34 | 4.33E-04 |
| C15orf48 | chromosome 15 open reading frame 48 | 3.12 | 2.30E-10 | 17.86 | 6.66E-06 |
| C1orf116 | chromosome 1 open reading frame 116 | 1.59 | 5.12E-06 | 5.29 | 1.00E-24 |
| CADM1 | cell adhesion molecule 1 | 0.60 | 1.34E-05 | 0.27 | 1.13E-46 |
| CASP6 | caspase 6, apoptosis-related cysteine peptidase | 0.73 | 4.37E-04 | 0.53 | 2.00E-04 |
| CBL | Cbl proto-oncogene, E3 ubiquitin protein ligase | 1.55 | 1.86E-04 | 1.59 | 2.35E-06 |
| CCDC92 | coiled-coil domain containing 92 | 0.60 | 8.68E-06 | 1.81 | 2.00E-05 |
| CCNB1IP1 | cyclin B1 interacting protein 1, E3 ubiquitin protein ligase | 1.51 | 2.44E-05 | 1.87 | 1.95E-11 |
| CD44 | CD44 molecule (Indian blood group) | 0.52 | 5.75E-08 | 8.72 | 1.45E-08 |
| CD68 | CD68 molecule | 0.50 | 6.78E-08 | 2.02 | 4.23E-06 |
| CDA | cytidine deaminase | 7.63 | 3.88E-13 | 9.40 | 4.53E-44 |
| CDC16 | cell division cycle 16 | 0.67 | 1.78E-04 | 1.51 | 1.20E-04 |
| CDCA7 | cell division cycle associated 7 | 0.67 | 9.88E-05 | 0.26 | 7.37E-21 |
| CDKN1A | cyclin-dependent kinase inhibitor 1A (p21, Cip1) | 1.67 | 2.80E-06 | 2.18 | 3.23E-23 |
| CFB | complement factor B | 2.85 | 3.53E-11 | 0.35 | 1.17E-32 |
| CHRNA5 | cholinergic receptor, nicotinic, alpha 5 (neuronal) | 0.68 | 1.23E-04 | 1.93 | 2.41E-05 |
| CHRNB1 | cholinergic receptor, nicotinic, beta 1 (muscle) | 1.71 | 2.11E-06 | 1.83 | 2.83E-07 |
| COL18A1 | collagen, type XVIII, alpha 1 | 0.69 | 9.36E-05 | 0.61 | 2.95E-09 |
| COMT | catechol-O-methyltransferase | 0.67 | 5.04E-05 | 0.49 | 1.60E-11 |
| COQ7 | coenzyme Q7 homolog, ubiquinone (yeast) | 0.74 | 4.17E-04 | 0.49 | 1.61E-06 |
| CRCP | CGRP receptor component | 0.66 | 1.41E-04 | 1.98 | 2.09E-07 |
| CREBRF | CREB3 regulatory factor | 1.50 | 8.79E-05 | 3.36 | 4.14E-24 |
| CREG1 | cellular repressor of E1A-stimulated genes 1 | 0.69 | 3.29E-04 | 0.44 | 3.75E-06 |
| CSRNP1 | cysteine-serine-rich nuclear protein 1 | 0.61 | 2.91E-05 | 3.15 | 3.31E-24 |
| CTGF | connective tissue growth factor | 0.61 | 1.27E-05 | 1.69 | 1.54E-10 |

**Supplementary Table 3.** Common 252 genes regulated by NR2E3 deletion in both MCF-7 and HepG2 cells.

| SYMBOL | Description | MCF-7 | | HepG2 | |
|---|---|---|---|---|---|
| | | Fold Change | P value | Fold Change | P value |
| CTPS2 | CTP synthase 2 | 0.72 | 4.10E-04 | 0.35 | 2.25E-16 |
| CTSB | cathepsin B | 0.61 | 3.91E-06 | 1.83 | 5.11E-16 |
| CUL4A | cullin 4A | 0.70 | 3.08E-04 | 1.40 | 1.26E-04 |
| CXCL8 | chemokine (C-X-C motif) ligand 8 | 2.72 | 1.73E-10 | 21.01 | 1.10E-197 |
| CXCR4 | chemokine (C-X-C motif) receptor 4 | 0.57 | 9.12E-07 | 20.12 | 1.12E-100 |
| CYB5A | cytochrome b5 type A (microsomal) | 1.58 | 1.39E-04 | 0.45 | 2.75E-14 |
| CYP1A1 | cytochrome P450, family 1, subfamily A, polypeptide 1 | 1.57 | 1.34E-05 | 1.88 | 7.49E-07 |
| DAPK3 | death-associated protein kinase 3 | 1.47 | 3.96E-05 | 2.08 | 1.52E-14 |
| DDB2 | damage-specific DNA binding protein 2, 48kDa | 1.62 | 6.33E-06 | 0.69 | 3.45E-04 |
| DDIT3 | DNA-damage-inducible transcript 3 | 1.79 | 3.17E-07 | 10.73 | 2.36E-36 |
| DHRS2 | dehydrogenase/reductase (SDR family) member 2 | 1.43 | 9.13E-05 | 0.58 | 7.79E-10 |
| DNAJB6 | DnaJ (Hsp40) homolog, subfamily B, member 6 | 0.66 | 4.22E-05 | 2.48 | 5.98E-26 |
| DRAM1 | DNA-damage regulated autophagy modulator 1 | 1.46 | 9.75E-05 | 0.44 | 4.66E-06 |
| E2F2 | E2F transcription factor 2 | 0.62 | 8.30E-06 | 0.35 | 5.21E-05 |
| EDN1 | endothelin 1 | 1.59 | 1.25E-05 | 3.19 | 3.32E-11 |
| EGLN3 | egl-9 family hypoxia-inducible factor 3 | 0.74 | 3.66E-04 | 3.57 | 6.13E-09 |
| EIF2AK2 | eukaryotic translation initiation factor 2-alpha kinase 2 | 2.71 | 1.43E-08 | 1.58 | 3.85E-04 |
| ERBB3 | v-erb-b2 avian erythroblastic leukemia viral oncogene homolog 3 | 0.65 | 2.52E-05 | 0.23 | 3.94E-62 |
| ERP27 | endoplasmic reticulum protein 27 | 2.46 | 2.98E-09 | 0.15 | 1.34E-30 |
| ERRFI1 | ERBB receptor feedback inhibitor 1 | 1.44 | 9.70E-05 | 1.99 | 1.11E-18 |
| FAH | fumarylacetoacetate hydrolase (fumarylacetoacetase) | 1.48 | 3.42E-05 | 0.61 | 1.70E-05 |
| FAM178A | family with sequence similarity 178, member A | 0.67 | 3.03E-04 | 0.28 | 8.29E-30 |
| FAM217B | family with sequence similarity 217, member B | 0.70 | 1.45E-04 | 0.62 | 2.64E-04 |
| FAM3C | family with sequence similarity 3, member C | 0.64 | 1.08E-04 | 1.95 | 5.49E-06 |
| FAM46A | family with sequence similarity 46, member A | 0.70 | 9.94E-05 | 1.99 | 1.36E-10 |
| FAM73A | family with sequence similarity 73, member A | 0.61 | 1.19E-05 | 1.56 | 2.11E-04 |
| FAT1 | FAT atypical cadherin 1 | 0.59 | 2.34E-06 | 0.72 | 1.46E-05 |
| FBXO32 | F-box protein 32 | 1.41 | 1.17E-04 | 14.52 | 1.75E-26 |
| FKBP4 | FK506 binding protein 4, 59kDa | 0.67 | 3.57E-05 | 2.24 | 2.73E-09 |
| FKTN | fukutin | 0.68 | 1.91E-04 | 0.45 | 2.48E-07 |
| FOSB | FBJ murine osteosarcoma viral oncogene homolog B | 0.56 | 2.19E-06 | 25.89 | 1.30E-95 |
| FTH1P3 | ferritin, heavy polypeptide 1 pseudogene 3 | 1.54 | 2.28E-04 | 1.38 | 4.98E-05 |
| GABARAPL1 | GABA(A) receptor-associated protein like 1 | 1.70 | 1.08E-06 | 3.91 | 2.64E-38 |
| GADD45A | growth arrest and DNA-damage-inducible, alpha | 2.01 | 1.41E-08 | 3.53 | 5.10E-29 |
| GAMT | guanidinoacetate N-methyltransferase | 1.38 | 3.19E-04 | 0.53 | 1.26E-04 |
| GDAP1 | ganglioside induced differentiation associated protein 1 | 0.72 | 3.76E-04 | 0.34 | 9.54E-05 |
| GDF15 | growth differentiation factor 15 | 4.43 | 9.89E-14 | 2.81 | 2.76E-37 |
| GJC1 | gap junction protein, gamma 1, 45kDa | 0.70 | 2.85E-04 | 4.76 | 6.72E-36 |
| GOLT1A | golgi transport 1A | 1.47 | 2.44E-04 | 3.78 | 5.62E-19 |
| GPD1L | glycerol-3-phosphate dehydrogenase 1-like | 0.63 | 2.25E-05 | 0.36 | 4.98E-16 |
| GPER1 | G protein-coupled estrogen receptor 1 | 0.58 | 6.83E-07 | 0.54 | 7.92E-05 |
| GPX3 | glutathione peroxidase 3 (plasma) | 0.65 | 8.62E-05 | 0.27 | 8.00E-48 |
| GRAMD3 | GRAM domain containing 3 | 1.48 | 3.98E-05 | 2.61 | 3.45E-11 |
| GRB7 | growth factor receptor-bound protein 7 | 1.53 | 3.61E-05 | 0.43 | 2.28E-10 |
| GTF2H1 | general transcription factor IIH, polypeptide 1, 62kDa | 0.69 | 8.22E-05 | 1.62 | 2.32E-05 |
| GTF2IP1 | general transcription factor IIi, pseudogene 1 | 0.68 | 1.41E-04 | 0.47 | 2.56E-08 |
| HCFC1R1 | host cell factor C1 regulator 1 (XPO1 dependent) | 1.89 | 7.32E-05 | 1.75 | 8.81E-05 |
| HLA-G | major histocompatibility complex, class I, G | 1.49 | 1.43E-04 | 2.32 | 3.63E-04 |
| HMGA1 | high mobility group AT-hook 1 | 1.53 | 5.68E-05 | 2.81 | 1.57E-31 |
| HMGB1 | high mobility group box 1 | 0.68 | 1.47E-04 | 0.52 | 1.16E-14 |
| HMGB3 | high mobility group box 3 | 0.71 | 1.47E-04 | 0.32 | 7.58E-18 |
| HMOX1 | heme oxygenase (decycling) 1 | 2.39 | 3.92E-08 | 10.44 | 8.60E-55 |
| HNMT | histamine N-methyltransferase | 1.81 | 9.75E-04 | 0.45 | 3.63E-14 |
| HNRNPA1P10 | heterogeneous nuclear ribonucleoprotein A1 pseudogene 10 | 0.72 | 4.42E-04 | 0.35 | 3.35E-42 |
| HNRNPAB | heterogeneous nuclear ribonucleoprotein A/B | 0.64 | 1.48E-05 | 0.33 | 2.56E-33 |
| HRK | harakiri, BCL2 interacting protein | 1.52 | 2.55E-04 | 20.88 | 5.49E-10 |
| HSH2D | hematopoietic SH2 domain containing | 1.50 | 3.83E-05 | 3.76 | 2.10E-05 |
| HSPA12A | heat shock 70kDa protein 12A | 0.71 | 1.27E-04 | 0.58 | 3.34E-04 |

**Supplementary Table 3.** Common 252 genes regulated by NR2E3 deletion in both MCF-7 and HepG2 cells.

| SYMBOL | Description | MCF-7 | | HepG2 | |
|---|---|---|---|---|---|
| | | Fold Change | P value | Fold Change | P value |
| HYAL1 | hyaluronoglucosaminidase 1 | 1.53 | 1.72E-05 | 0.61 | 3.77E-06 |
| ID2 | inhibitor of DNA binding 2, dominant negative helix-loop-helix protein | 0.71 | 1.81E-04 | 0.37 | 2.69E-30 |
| IFITM3 | interferon induced transmembrane protein 3 | 4.64 | 6.49E-10 | 0.67 | 6.72E-05 |
| IFNAR1 | interferon (alpha, beta and omega) receptor 1 | 0.56 | 1.84E-06 | 0.67 | 2.04E-04 |
| IFT122 | intraflagellar transport 122 | 0.64 | 7.80E-06 | 0.53 | 8.70E-05 |
| IGSF1 | immunoglobulin superfamily, member 1 | 1.57 | 5.27E-06 | 0.53 | 5.24E-09 |
| IL17RB | interleukin 17 receptor B | 0.62 | 1.09E-05 | 0.52 | 3.47E-10 |
| IL17RD | interleukin 17 receptor D | 0.60 | 3.55E-06 | 2.55 | 2.51E-06 |
| IL18 | interleukin 18 | 0.58 | 3.53E-06 | 3.37 | 3.04E-36 |
| INHBB | inhibin, beta B | 1.67 | 1.82E-05 | 0.63 | 3.75E-05 |
| INTS6 | integrator complex subunit 6 | 0.72 | 2.48E-04 | 1.43 | 4.05E-04 |
| IRF9 | interferon regulatory factor 9 | 6.53 | 2.47E-15 | 2.11 | 1.03E-06 |
| ITGA5 | integrin, alpha 5 (fibronectin receptor, alpha polypeptide) | 1.47 | 1.05E-04 | 21.12 | 6.85E-137 |
| JAK2 | Janus kinase 2 | 0.74 | 3.73E-04 | 1.91 | 7.93E-05 |
| KIAA0513 | KIAA0513 | 1.78 | 1.42E-06 | 3.19 | 2.74E-11 |
| KIF15 | kinesin family member 15 | 0.67 | 1.56E-04 | 0.29 | 9.77E-15 |
| KLF2 | Kruppel-like factor 2 | 0.60 | 4.25E-06 | 33.57 | 8.83E-11 |
| KRT15 | keratin 15 | 0.60 | 1.13E-05 | 21.77 | 2.35E-57 |
| KRT80 | keratin 80 | 1.81 | 3.35E-06 | 1.49 | 5.01E-06 |
| LAMC2 | laminin, gamma 2 | 1.37 | 2.79E-04 | 82.68 | 1.44E-21 |
| LCP1 | lymphocyte cytosolic protein 1 (L-plastin) | 2.13 | 6.55E-09 | 5.32 | 3.21E-33 |
| LMCD1 | LIM and cysteine-rich domains 1 | 1.68 | 3.17E-06 | 2.33 | 8.75E-13 |
| MACF1 | microtubule-actin crosslinking factor 1 | 0.71 | 4.79E-04 | 1.71 | 4.78E-12 |
| MALT1 | mucosa associated lymphoid tissue lymphoma translocation gene 1 | 0.73 | 4.59E-04 | 0.49 | 1.09E-11 |
| MB21D2 | Mab-21 domain containing 2 | 1.52 | 3.54E-05 | 2.47 | 1.16E-04 |
| MBD4 | methyl-CpG binding domain protein 4 | 0.67 | 1.50E-04 | 0.62 | 1.22E-04 |
| MBNL3 | muscleblind-like splicing regulator 3 | 0.67 | 4.18E-05 | 0.18 | 5.59E-102 |
| MCM10 | minichromosome maintenance complex component 10 | 0.73 | 3.14E-04 | 0.28 | 6.90E-14 |
| MCM8 | minichromosome maintenance complex component 8 | 0.63 | 4.31E-05 | 0.58 | 5.40E-07 |
| MLEC | malectin | 0.73 | 4.72E-04 | 0.40 | 5.72E-27 |
| MTSS1 | metastasis suppressor 1 | 0.62 | 4.03E-06 | 0.50 | 1.40E-07 |
| MVP | major vault protein | 1.79 | 7.40E-06 | 1.71 | 4.42E-07 |
| MYEOV | myeloma overexpressed | 1.61 | 5.41E-06 | 7.91 | 9.68E-36 |
| MYH9 | myosin, heavy chain 9, non-muscle | 1.40 | 1.61E-04 | 1.31 | 2.54E-04 |
| MYO1B | myosin IB | 1.86 | 7.24E-07 | 0.49 | 9.95E-09 |
| NBPF1 | neuroblastoma breakpoint family, member 1 | 0.67 | 1.65E-04 | 1.34 | 4.47E-04 |
| NFKB2 | nuclear factor of kappa light polypeptide gene enhancer in B-cells 2 (p49/p100) | 1.51 | 2.64E-05 | 2.54 | 3.41E-18 |
| NRP1 | neuropilin 1 | 1.86 | 1.48E-06 | 0.66 | 6.22E-06 |
| NTN4 | netrin 4 | 2.39 | 1.18E-09 | 1.61 | 4.09E-06 |
| NUAK2 | NUAK family, SNF1-like kinase, 2 | 1.54 | 1.25E-04 | 3.70 | 2.45E-24 |
| OAS1 | 2'-5'-oligoadenylate synthetase 1, 40/46kDa | 4.56 | 1.67E-13 | 5.59 | 6.98E-40 |
| OAS3 | 2'-5'-oligoadenylate synthetase 3, 100kDa | 6.44 | 1.47E-14 | 1.46 | 2.86E-04 |
| OPTN | optineurin | 1.41 | 1.75E-04 | 3.34 | 3.27E-40 |
| PANK1 | pantothenate kinase 1 | 0.49 | 3.02E-08 | 0.41 | 8.97E-08 |
| PAPOLA | poly(A) polymerase alpha | 0.73 | 4.70E-04 | 0.64 | 2.32E-07 |
| PARP14 | poly (ADP-ribose) polymerase family, member 14 | 3.72 | 6.56E-13 | 0.67 | 1.88E-04 |
| PBX2 | pre-B-cell leukemia homeobox 2 | 1.50 | 3.61E-04 | 0.41 | 4.29E-17 |
| PCYOX1 | prenylcysteine oxidase 1 | 0.68 | 4.72E-05 | 0.53 | 8.08E-15 |
| PHLDA3 | pleckstrin homology-like domain, family A, member 3 | 1.61 | 4.30E-06 | 1.76 | 1.76E-05 |
| PIK3R1 | phosphoinositide-3-kinase, regulatory subunit 1 (alpha) | 0.58 | 1.05E-06 | 0.55 | 2.55E-08 |
| PIP4K2A | phosphatidylinositol-5-phosphate 4-kinase, type II, alpha | 0.67 | 5.02E-05 | 1.69 | 9.73E-07 |
| PKD1L2 | polycystic kidney disease 1-like 2 | 1.44 | 6.25E-05 | 4.96 | 3.36E-20 |
| PKIA | protein kinase (cAMP-dependent, catalytic) inhibitor alpha | 0.68 | 4.88E-05 | 34.47 | 1.20E-07 |
| PLXND1 | plexin D1 | 0.74 | 4.50E-04 | 0.55 | 4.62E-13 |
| PMP22 | peripheral myelin protein 22 | 0.50 | 6.53E-08 | 4.20 | 2.83E-24 |
| PODXL | podocalyxin-like | 0.60 | 1.92E-06 | 4.33 | 2.82E-12 |
| PROSER2 | proline and serine rich 2 | 1.64 | 8.02E-06 | 2.96 | 4.68E-18 |
| PSG4 | pregnancy specific beta-1-glycoprotein 4 | 1.81 | 4.15E-07 | 8.37 | 4.03E-04 |

**Supplementary Table 3.** Common 252 genes regulated by NR2E3 deletion in both MCF-7 and HepG2 cells.

| SYMBOL | Description | MCF-7 | | HepG2 | |
|---|---|---|---|---|---|
| | | Fold Change | P value | Fold Change | P value |
| PSME2 | proteasome (prosome, macropain) activator subunit 2 (PA28 beta) | 1.96 | 6.77E-06 | 0.63 | 3.26E-06 |
| PTPN11 | protein tyrosine phosphatase, non-receptor type 11 | 0.52 | 2.99E-07 | 0.36 | 7.79E-31 |
| PVRL3 | poliovirus receptor-related 3 | 0.57 | 3.33E-07 | 0.52 | 1.54E-05 |
| PYGL | phosphorylase, glycogen, liver | 1.43 | 3.21E-04 | 0.58 | 9.34E-10 |
| PZP | pregnancy-zone protein | 0.69 | 5.38E-05 | 0.26 | 3.79E-09 |
| RAB11FIP3 | RAB11 family interacting protein 3 (class II) | 0.70 | 1.88E-04 | 0.62 | 6.77E-06 |
| RAB22A | RAB22A, member RAS oncogene family | 0.51 | 2.89E-08 | 1.39 | 4.36E-04 |
| RAB29 | RAB29, member RAS oncogene family | 1.64 | 4.37E-06 | 1.81 | 1.14E-05 |
| RAB4A | RAB4A, member RAS oncogene family | 0.67 | 5.19E-05 | 0.46 | 2.98E-11 |
| RAD51 | RAD51 recombinase | 0.72 | 3.91E-04 | 0.43 | 1.80E-04 |
| RCC1 | regulator of chromosome condensation 1 | 1.60 | 1.06E-05 | 0.61 | 1.54E-05 |
| RIT1 | Ras-like without CAAX 1 | 1.41 | 1.48E-04 | 4.21 | 1.57E-36 |
| RNF213 | ring finger protein 213 | 0.55 | 2.01E-06 | 0.75 | 2.43E-04 |
| RRAS | related RAS viral (r-ras) oncogene homolog | 2.03 | 2.62E-07 | 3.57 | 6.40E-14 |
| S100A10 | S100 calcium binding protein A10 | 2.70 | 1.70E-07 | 3.32 | 1.24E-42 |
| S100A14 | S100 calcium binding protein A14 | 1.51 | 1.65E-04 | 2.29 | 5.19E-07 |
| SAT1 | spermidine/spermine N1-acetyltransferase 1 | 1.56 | 1.39E-05 | 2.22 | 6.42E-10 |
| SAYSD1 | SAYSVFN motif domain containing 1 | 0.60 | 3.00E-06 | 0.37 | 8.19E-07 |
| SEL1L3 | sel-1 suppressor of lin-12-like 3 (C. elegans) | 1.43 | 1.18E-04 | 2.83 | 2.41E-21 |
| SERPINE2 | serpin peptidase inhibitor, clade E (nexin, plasminogen activator inhibitor type 1), member 2 | 0.67 | 1.80E-05 | 1.40 | 2.19E-05 |
| SFXN2 | sideroflexin 2 | 0.58 | 7.60E-07 | 0.27 | 1.06E-06 |
| SGK1 | serum/glucocorticoid regulated kinase 1 | 0.53 | 4.56E-07 | 4.11 | 7.23E-28 |
| SHCBP1 | SHC SH2-domain binding protein 1 | 0.66 | 2.81E-05 | 0.17 | 5.26E-30 |
| SLC25A15 | solute carrier family 25 (mitochondrial carrier; ornithine transporter) member 15 | 0.68 | 4.32E-05 | 0.48 | 1.81E-08 |
| SLC25A25 | solute carrier family 25 (mitochondrial carrier; phosphate carrier), member 25 | 0.60 | 1.19E-06 | 2.37 | 1.92E-08 |
| SLC38A1 | solute carrier family 38, member 1 | 0.67 | 3.00E-04 | 1.52 | 3.89E-07 |
| SLC47A1 | solute carrier family 47 (multidrug and toxin extrusion), member 1 | 0.59 | 2.09E-05 | 0.31 | 3.34E-33 |
| SLX1A | SLX1 structure-specific endonuclease subunit homolog A (S. cerevisiae) | 1.40 | 4.14E-04 | 1.49 | 1.78E-05 |
| SMARCB1 | SWI/SNF related, matrix associated, actin dependent regulator of chromatin, subfamily b, member 1 | 0.73 | 3.10E-04 | 0.66 | 1.80E-05 |
| SNAPC1 | small nuclear RNA activating complex, polypeptide 1, 43kDa | 0.51 | 9.18E-08 | 1.97 | 9.08E-05 |
| SNX10 | sorting nexin 10 | 0.71 | 2.26E-04 | 0.68 | 3.49E-04 |
| SP100 | SP100 nuclear antigen | 2.11 | 8.55E-08 | 1.50 | 9.41E-05 |
| SP110 | SP110 nuclear body protein | 4.13 | 7.54E-13 | 2.10 | 2.95E-10 |
| SPHAR | S-phase response (cyclin related) | 0.68 | 5.57E-05 | 0.23 | 1.11E-04 |
| SPINT1 | serine peptidase inhibitor, Kunitz type 1 | 1.70 | 4.21E-05 | 4.24 | 3.60E-40 |
| SPRY1 | sprouty homolog 1, antagonist of FGF signaling (Drosophila) | 1.54 | 2.88E-05 | 0.52 | 3.96E-05 |
| STAT1 | signal transducer and activator of transcription 1, 91kDa | 4.88 | 4.48E-13 | 0.56 | 2.45E-10 |
| STK19 | serine/threonine kinase 19 | 1.41 | 3.65E-04 | 1.89 | 5.47E-06 |
| STK40 | serine/threonine kinase 40 | 1.40 | 4.52E-04 | 1.97 | 4.38E-14 |
| STRA6 | stimulated by retinoic acid 6 | 1.41 | 1.27E-04 | 0.13 | 4.60E-06 |
| SYBU | syntabulin (syntaxin-interacting) | 0.65 | 1.70E-05 | 2.97 | 3.11E-08 |
| TAF9B | TAF9B RNA polymerase II, TATA box binding protein (TBP)-associated factor, 31kDa | 0.69 | 1.85E-04 | 0.52 | 4.36E-05 |
| TAGLN2 | transgelin 2 | 1.44 | 1.78E-04 | 1.67 | 1.57E-08 |
| TEX19 | testis expressed 19 | 3.68 | 2.62E-12 | 14.96 | 1.37E-07 |
| TGM2 | transglutaminase 2 | 1.68 | 1.74E-06 | 1.87 | 1.35E-15 |
| TIMP2 | TIMP metallopeptidase inhibitor 2 | 1.55 | 1.80E-05 | 1.52 | 1.35E-07 |
| TIPARP | TCDD-inducible poly(ADP-ribose) polymerase | 0.64 | 3.92E-05 | 2.06 | 1.56E-08 |
| TMEM140 | transmembrane protein 140 | 1.76 | 1.77E-06 | 0.63 | 1.06E-04 |
| TMEM181 | transmembrane protein 181 | 0.64 | 2.67E-05 | 0.39 | 2.02E-27 |
| TMEM241 | transmembrane protein 241 | 0.71 | 3.56E-04 | 2.23 | 2.74E-05 |
| TMEM254 | transmembrane protein 254 | 0.55 | 1.00E-06 | 0.30 | 7.10E-08 |
| TMSB4X | thymosin beta 4, X-linked | 1.50 | 1.34E-04 | 2.21 | 6.16E-17 |
| TNFRSF12A | tumor necrosis factor receptor superfamily, member 12A | 2.16 | 5.28E-07 | 2.46 | 2.50E-21 |
| TNFSF14 | tumor necrosis factor (ligand) superfamily, member 14 | 0.56 | 6.97E-07 | 4.53 | 8.60E-12 |
| TNFSF15 | tumor necrosis factor (ligand) superfamily, member 15 | 0.62 | 4.81E-06 | 11.84 | 1.05E-18 |

**Supplementary Table 3.** Common 252 genes regulated by NR2E3 deletion in both MCF-7 and HepG2 cells.

| SYMBOL | Description | MCF-7 | | HepG2 | |
|---|---|---|---|---|---|
| | | Fold Change | P value | Fold Change | P value |
| TNS3 | tensin 3 | 1.62 | 3.98E-06 | 0.61 | 1.13E-08 |
| TRAFD1 | TRAF-type zinc finger domain containing 1 | 1.43 | 8.27E-05 | 1.62 | 4.41E-04 |
| TRAK2 | trafficking protein, kinesin binding 2 | 0.59 | 1.47E-06 | 1.48 | 1.48E-04 |
| TRIB1 | tribbles pseudokinase 1 | 0.65 | 7.35E-05 | 2.06 | 4.21E-16 |
| TSC22D2 | TSC22 domain family, member 2 | 1.46 | 5.25E-05 | 3.29 | 1.37E-25 |
| TSPAN1 | tetraspanin 1 | 1.61 | 2.00E-05 | 3.00 | 8.39E-27 |
| TXNIP | thioredoxin interacting protein | 1.73 | 1.71E-06 | 0.23 | 3.43E-20 |
| UAP1L1 | UDP-N-acetylglucosamine pyrophosphorylase 1 like 1 | 0.68 | 6.40E-05 | 1.91 | 4.76E-07 |
| UBD | ubiquitin D | 2.86 | 3.09E-10 | 0.32 | 1.31E-28 |
| UBE2B | ubiquitin-conjugating enzyme E2B | 0.68 | 8.86E-05 | 1.71 | 1.31E-06 |
| UBE2Q1 | ubiquitin-conjugating enzyme E2Q family member 1 | 0.63 | 1.96E-05 | 0.52 | 8.93E-06 |
| UBE2Z | ubiquitin-conjugating enzyme E2Z | 0.67 | 2.04E-05 | 0.66 | 1.10E-05 |
| UHMK1 | U2AF homology motif (UHM) kinase 1 | 0.66 | 3.91E-05 | 1.40 | 1.67E-04 |
| UHRF1 | ubiquitin-like with PHD and ring finger domains 1 | 0.70 | 2.16E-04 | 0.24 | 1.06E-23 |
| VAV3 | vav 3 guanine nucleotide exchange factor | 0.69 | 1.50E-04 | 0.22 | 7.19E-10 |
| WFS1 | Wolfram syndrome 1 (wolframin) | 0.65 | 1.20E-04 | 0.40 | 1.13E-08 |
| WRAP53 | WD repeat containing, antisense to TP53 | 0.69 | 1.56E-04 | 0.48 | 6.17E-07 |
| XPO4 | exportin 4 | 0.73 | 3.71E-04 | 0.48 | 6.22E-05 |
| ZNF185 | zinc finger protein 185 (LIM domain) | 0.70 | 1.27E-04 | 3.07 | 5.98E-22 |
| ZNF394 | zinc finger protein 394 | 0.70 | 1.23E-04 | 1.93 | 2.52E-04 |
| ZNF430 | zinc finger protein 430 | 0.66 | 1.59E-05 | 2.65 | 1.90E-06 |
| ZNF544 | zinc finger protein 544 | 0.65 | 2.61E-05 | 0.42 | 9.19E-06 |
| ZNFX1 | zinc finger, NFX1-type containing 1 | 2.02 | 2.24E-08 | 1.89 | 1.84E-11 |

**Supplementary Table. 4.** Major signaling pathways associated with NR2E3 gene networks in HepG2 cells by Wikipathway analysis.

| GeneSet Name | GeneSet ID | Enrichment Ratio | rawP | adjP Genes |
|---|---|---|---|---|
| Cell cycle | WP179 | 4.71 | 2.43E-20 | 1.24E-18 |
| Adipogenesis | WP236 | 4.24 | 2.87E-19 | 9.76E-18 |
| DNA Replication | WP466 | 7.24 | 9.77E-19 | 2.85E-17 |
| G1 to S cell cycle control | WP45 | 4.68 | 1.67E-14 | 4.26E-13 |
| Signaling Pathways in Glioblastoma | WP2261 | 4.42 | 4.59E-14 | 8.66E-13 |
| Apoptosis | WP254 | 4.04 | 9.45E-13 | 1.61E-11 |
| Complement and Coagulation Cascades | WP558 | 5.08 | 1.03E-11 | 1.24E-10 |
| miRNA regulation of DNA Damage Response | WP1530 | 4.26 | 1.10E-11 | 1.25E-10 |
| DNA damage response | WP707 | 4.28 | 2.22E-11 | 2.38E-10 |
| Statin Pathway | WP430 | 5.63 | 7.00E-09 | 4.76E-08 |
| Selenium Metabolism and Selenoproteins | WP28 | 4.37 | 1.38E-08 | 8.53E-08 |
| IL-4 signaling pathway | WP395 | 4.01 | 1.46E-08 | 8.76E-08 |
| Hypertrophy Model | WP516 | 6.19 | 2.11E-07 | 1.02E-06 |
| Blood Clotting Cascade | WP272 | 5.2 | 6.98E-07 | 3.16E-06 |
| G13 Signaling Pathway | WP524 | 4.26 | 1.58E-06 | 6.86E-06 |
| SREBF and miR33 in cholesterol and lipid homeostasis | WP2011 | 6.33 | 2.19E-06 | 9.31E-06 |
| **AhR pathway** | WP2100 | 4.04 | 3.29E-06 | 1.37E-05 |
| Complement Activation, Classical Pathway | WP545 | 5.96 | 4.28E-06 | 1.71E-05 |
| Mismatch repair | WP531 | 6.43 | 7.08E-06 | 2.78E-05 |
| ID signaling pathway | WP53 | 6 | 1.40E-05 | 4.76E-05 |
| Triacylglyceride Synthesis | WP325 | 4.69 | 1.84E-05 | 6.15E-05 |
| Fatty Acid Biosynthesis | WP357 | 4.27 | 2.04E-05 | 6.61E-05 |
| Nucleotide Metabolism | WP404 | 5.07 | 2.31E-05 | 7.36E-05 |
| Glutathione metabolism | WP100 | 4.5 | 2.83E-05 | 8.62E-05 |
| Oxidative Stress | WP408 | 4.13 | 2.96E-05 | 8.84E-05 |
| Adipocyte TarBase | WP2001 | 4.61 | 5.80E-05 | 0.0002 |
| TCA Cycle | WP78 | 4.5 | 0.0002 | 0.0005 |
| IL-7 signaling pathway | WP205 | 4.05 | 0.0002 | 0.0005 |
| Keap1-Nrf2 Pathway | WP3 | 4.64 | 0.0004 | 0.0009 |
| Urea cycle and metabolism of amino groups | WP497 | 4.09 | 0.0004 | 0.0009 |
| TP53 network | WP1742 | 4.15 | 0.0008 | 0.0016 |
| **Benzo(a)pyrene metabolism** | WP696 | 5.12 | 0.0016 | 0.003 |
| Glial Cell Differentiation | WP2276 | 6.43 | 0.0017 | 0.0031 |
| E2F-MIRHG1 feedback-loop | WP1537 | 8.44 | 0.0026 | 0.0047 |
| Glycoprotein VI platelet signaling | WP1970 | 11.26 | 0.0079 | 0.0128 |
| Nifedipine Activity | WP259 | 5.63 | 0.0114 | 0.018 |
| IL12 SIGNALING PATHWAY | WP2111 | 4.09 | 0.0123 | 0.0192 |
| Integrated Cancer pathway | WP1971 | 7.5 | 0.0223 | 0.0335 |
| Methylation Pathways | WP704 | 4.22 | 0.0279 | 0.0398 |
| Pentose Phosphate Pathway | WP134 | 4.22 | 0.0279 | 0.0398 |

**Supplementary Table. 5.** Major signaling pathways associated with NR2E3 gene netwroks in MCF-7 cells by Wikipathway analysis .

| GeneSet Name | GeneSet ID | Enrichment Ratio | rawP | adjP |
|---|---|---|---|---|
| B Cell Receptor Signaling Pathway | WP23 | 6.13 | 0.0043 | 0.0091 |
| TWEAK Signaling Pathway | WP2036 | 9.35 | 0.0041 | 0.0089 |
| Alpha 6 Beta 4 signaling pathway | WP244 | 10.48 | 0.003 | 0.0067 |
| Type III interferon signaling | WP2113 | 24.94 | 0.0028 | 0.0064 |
| Insulin Signaling | WP481 | 5.36 | 0.0026 | 0.0061 |
| Leukocyte TarBase | WP2003 | 5.46 | 0.0024 | 0.0058 |
| Regulation of toll-like receptor signaling pathway | WP1449 | 5.67 | 0.002 | 0.005 |
| **AhR pathway** | WP2100 | 13.43 | 0.0015 | 0.0039 |
| Oncostatin M Signaling Pathway | WP2358 | 8.22 | 0.0015 | 0.0039 |
| IL17 signaling pathway | WP2112 | 13.78 | 0.0014 | 0.0038 |
| Leptin signaling pathway | WP2034 | 8.62 | 0.0012 | 0.0034 |
| Methylation Pathways | WP704 | 43.65 | 0.0009 | 0.0026 |
| miRNA regulation of DNA Damage Response | WP1530 | 9.44 | 0.0009 | 0.0026 |
| DNA damage response | WP707 | 9.84 | 0.0008 | 0.0025 |
| **Oxidative Stress** | WP408 | 17.46 | 0.0007 | 0.0023 |
| metapathway biotransformation | WP702 | 5.92 | 0.0006 | 0.002 |
| TCR Signaling Pathway | WP69 | 8.24 | 0.0004 | 0.0014 |
| Cell cycle | WP179 | 7.94 | 0.0004 | 0.0014 |
| IL-3 Signaling Pathway | WP286 | 12.93 | 0.0003 | 0.0012 |
| IL-6 signaling pathway | WP364 | 12.04 | 0.0003 | 0.0012 |
| angiogenesis overview | WP1993 | 11.05 | 9.44E-05 | 0.0008 |
| Signaling Pathways in Glioblastoma | WP2261 | 10.39 | 0.0001 | 0.0008 |
| Adipogenesis | WP236 | 8.06 | 0.0001 | 0.0008 |
| IL-5 signaling pathway | WP127 | 14.86 | 0.0002 | 0.0008 |
| Interleukin-11 Signaling Pathway | WP2332 | 14.25 | 0.0002 | 0.0008 |
| TSLP Signaling Pathway | WP2203 | 14.25 | 0.0002 | 0.0008 |
| IL-2 Signaling pathway | WP49 | 13.18 | 0.0002 | 0.0008 |
| Prolactin Signaling Pathway | WP2037 | 9.49 | 0.0002 | 0.0008 |
| Apoptosis | WP254 | 9.49 | 0.0002 | 0.0008 |
| Squamous cell TarBase | WP2006 | 6.98 | 0.0002 | 0.0008 |
| Epithelium TarBase | WP2002 | 4.62 | 0.0002 | 0.0008 |
| Muscle cell TarBase | WP2005 | 4.12 | 0.0002 | 0.0008 |
| EGF-EGFR Signaling Pathway | WP437 | 7.11 | 6.50E-05 | 0.0007 |
| Lymphocyte TarBase | WP2004 | 3.93 | 6.71E-05 | 0.0007 |
| Senescence and Autophagy | WP615 | 8.73 | 7.04E-05 | 0.0007 |
| IL-4 signaling pathway | WP395 | 14.8 | 2.30E-05 | 0.0005 |
| Prostaglandin Synthesis and Regulation | WP98 | 22.53 | 2.93E-05 | 0.0005 |
| Type II interferon signaling (IFNG) | WP619 | 13.26 | 6.51E-06 | 0.0002 |
| Interferon type I | WP585 | 16.37 | 1.89E-06 | 9.61E-05 |
| Kit receptor signaling pathway | WP304 | 15.87 | 2.26E-06 | 9.61E-05 |

**Supplementary Table. 6.** Expression levels of NR2E3 in normal human liver tissue.

| Sample # | Diagnosis | NR2E3 staining Intensity |
|---|---|---|
| 1 | Normal | 40 |
| 2 | Normal | 100 |
| 3 | Normal | 100 |
| 4 | Normal | 100 |
| 5 | Normal | 100 |
| 6 | Normal | 60 |
| 7 | Normal | 100 |
| 8 | Normal | 100 |
| 9 | Normal | 100 |

**Supplementary Table. 7.** Expression levels of NR2E3 in cirrhotic human liver tissues.

| Sample # | Diagnosis | Associated lesion | NR2E3 staining Intensity |
|---|---|---|---|
| 1 | macronodular cirrhosis | Hepatocellular carcinoma | 100 |
| 2 | macronodular cirrhosis | Hepatocellular carcinoma | 100 |
| 3 | macronodular cirrhosis | Hepatocellular carcinoma | 100 |
| 4 | macronodular cirrhosis | Hepatocellular carcinoma | 100 |
| 5 | macronodular cirrhosis | Hepatocellular carcinoma | 100 |
| 6 | macronodular cirrhosis | Hepatocellular carcinoma | 100 |
| 7 | macronodular cirrhosis | Hepatocellular carcinoma | 100 |
| 8 | macronodular cirrhosis | Hepatocellular carcinoma | 100 |
| 9 | macronodular cirrhosis | Hepatocellular carcinoma | 100 |
| 10 | macro- and micronodular cirrhosis | Hepatocellular carcinoma | 100 |
| 11 | macronodular cirrhosis | Hepatocellular carcinoma | 80 |
| 12 | macronodular cirrhosis | Hepatocellular carcinoma | 80 |
| 13 | macro- and micronodular cirrhosis | Hepatocellular carcinoma | 80 |
| 14 | macronodular cirrhosis | Hepatocellular carcinoma | 70 |
| 15 | macronodular cirrhosis | Hepatocellular carcinoma | 60 |
| 16 | macronodular cirrhosis | Hepatocellular carcinoma | 60 |
| 17 | macronodular cirrhosis | Hepatocellular carcinoma | 60 |
| 18 | macronodular cirrhosis | Hepatocellular carcinoma | 60 |
| 19 | macro- and micronodular cirrhosis | Hepatocellular carcinoma | 60 |
| 20 | macro- and micronodular cirrhosis | Hepatocellular carcinoma | 60 |
| 21 | macronodular cirrhosis | Hepatocellular carcinoma | 50 |
| 22 | macronodular cirrhosis | Hepatocellular carcinoma | 50 |
| 23 | macronodular cirrhosis | Hepatocellular carcinoma | 30 |
| 24 | macro- and micronodular cirrhosis | Hepatocellular carcinoma | 30 |

**Supplementary Table. 8.** Clinical information and expression levels of NR2E3 in human liver tumor tissue samples (Hepatocellular carcinoma, HCC).

| Sample # | Diagnosis | T stage | N stage | M stage | NR2E3 staining Intensity (%) |
|---|---|---|---|---|---|
| 1 | HCC | T1 | N0 | M0 | 100 |
| 2 | HCC | T1 | N0 | M0 | 10 |
| 3 | HCC | T1 | N0 | M0 | 100 |
| 4 | HCC | T1 | N0 | M0 | 0 |
| 5 | HCC | T1 | N0 | M0 | 70 |
| 6 | HCC | T1 | N0 | M0 | 10 |
| 7 | HCC | T1 | N0 | M0 | 15 |
| 8 | HCC | T1 | N0 | M0 | 10 |
| 9 | HCC | T1 | N0 | M0 | 0 |
| 10 | HCC | T1 | N0 | M0 | 50 |
| 11 | HCC | T1 | N0 | M0 | 0 |
| 12 | HCC | T1 | N0 | M0 | 15 |
| 13 | HCC | T1 | N0 | M0 | 20 |
| 14 | HCC | T1 | N1 | M0 | 100 |
| 15 | HCC | T2 | N0 | M0 | 100 |
| 16 | HCC | T2 | N0 | M0 | 40 |
| 17 | HCC | T2 | N0 | M0 | 0 |
| 18 | HCC | T2 | N0 | M0 | 20 |
| 19 | HCC | T2 | N0 | M0 | 100 |
| 20 | HCC | T2 | N0 | M0 | 100 |
| 21 | HCC | T2 | N0 | M0 | 0 |
| 22 | HCC | T2 | N0 | M0 | 100 |
| 23 | HCC | T2 | N0 | M0 | 100 |
| 24 | HCC | T2 | N0 | M1 | 10 |
| 25 | HCC | T2 | Nx | M0 | 100 |
| 26 | HCC | T3 | N0 | M0 | 0 |
| 27 | HCC | T3 | N0 | M0 | 20 |
| 28 | HCC | T3 | N0 | M0 | 10 |
| 29 | HCC | T3 | N0 | M0 | 60 |
| 30 | HCC | T3 | N0 | M0 | 40 |
| 31 | HCC | T3 | N0 | M0 | 100 |
| 32 | HCC | T3a | N0 | M0 | 0 |
| 33 | HCC | T3a | N0 | M0 | 0 |
| 34 | HCC | T3a | N0 | M0 | 0 |
| 35 | HCC | T3a | N0 | M0 | 100 |
| 36 | HCC | T3a | N0 | M0 | 0 |
| 37 | HCC | T4 | Nx | M1 | 100 |
| 38 | HCC | T4 | N0 | M0 | 40 |
| 39 | HCC | T4 | N0 | M0 | 40 |
| 40 | HCC | T4 | N1 | M0 | 0 |
| 41 | HCC | TM | Nx | M1 | 60 |
| 42 | HCC | TM | Nx | M1 | 80 |
| 43 | HCC | TM | Nx | M1 | 20 |
| 44 | HCC | TM | Nx | M1 | 10 |
| 45 | HCC | TM | Nx | M1 | 100 |
| 46 | HCC | TM | N0 | M1 | 20 |
| 47 | HCC | TM | N1 | M0 | 70 |
| 48 | HCC | TM | Nx | M1 | 0 |
| 49 | HCC |  | N1 | M0 | 100 |
| 50 | HCC |  |  |  | 100 |

**Figure 3**

Fig. 3b

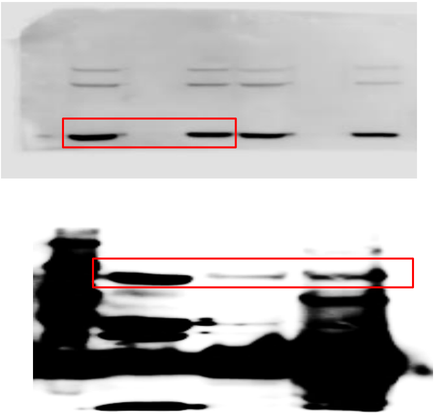

Fig. 3d

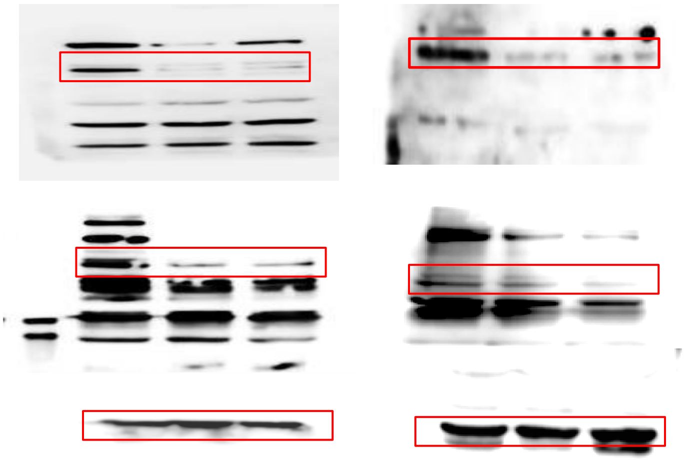

**Figure 4**

Fig. 4a

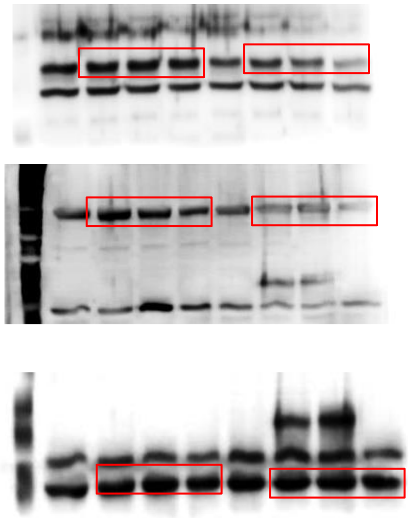

**Figure 5**

Fig. 5a

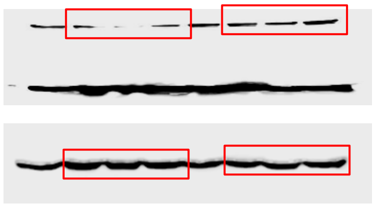

Fig. 5f

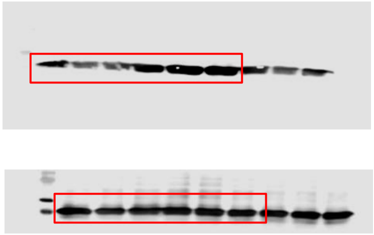

**Supplementary Fig. 10** Uncropped gels and western blots. The red boxes indicate the cropped regions.

**Supplementary Figure 1.**

Sup.Fig. 1a

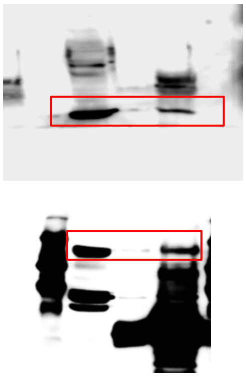

Sup.Fig. 1b

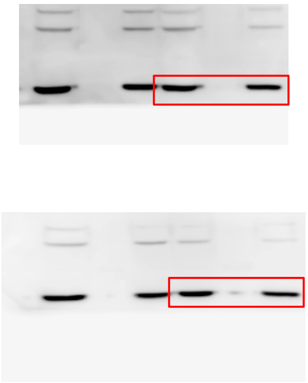

**Supplementary Figure 2.**

Sup.Fig. 2a

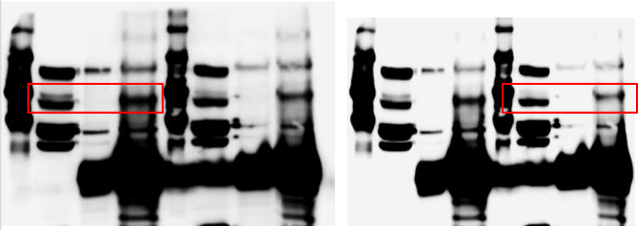

**Supplementary Figure 3.**

Sup.Fig. 3b

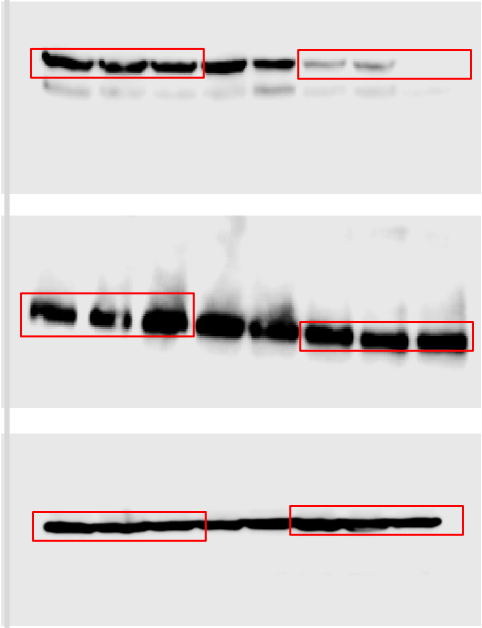

**Supplementary Figure 6.**

Sup.Fig. 6a

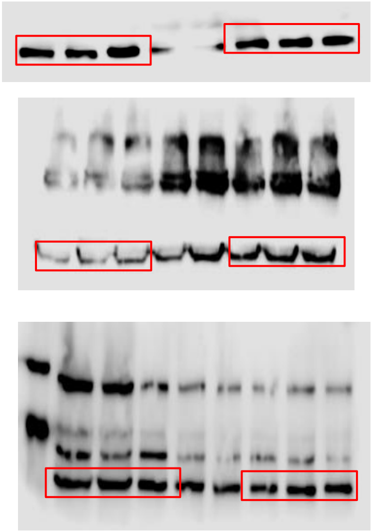

**Supplementary Figure 7.**

Sup.Fig. 7a

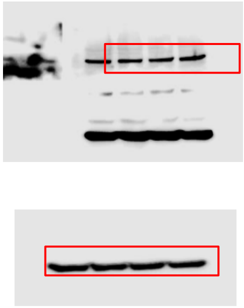

**Supplementary Fig. 11** Uncropped gels and western blots. The red boxes indicate the cropped regions
